# The methodology of design of satellite working mechanism of positive displacement machine

**DOI:** 10.1038/s41598-022-18093-z

**Published:** 2022-08-11

**Authors:** Pawel Sliwinski

**Affiliations:** Division of Hydraulics and Pneumatics, Faculty of Mechanical Engineering and Ship Technology, Gdansk University of Technology, ul. Gabriela Narutowicza 11/12, 80-233 Gdańsk, Poland

**Keywords:** Engineering, Mechanical engineering

## Abstract

In this paper is described a methodology of design of satellite mechanism consisting of two non-circular gears (externally toothed rotor and internally toothed curvature) and circular gears (satellites). In the presented methodology is assumed that the rotor pitch line is known, and the curvature pitch line is necessary to designate. The presented methodology applies to mechanisms for which the number of the curvature humps is at least one greater than the number of rotor humps. The selection of the number of gears and the number of teeth in gear and rotor and curvature is also presented. The methodology of calculating the position of the satellite center and the angle of its rotation in order to shape the teeth on the rotor and curvature is presented. The article is also showed different types of satellite mechanisms—satellite mechanisms with the different numbers of humps on the rotor and curvature. The technical parameters of the mechanism for the rotor pitch line described by the cosine function are also presented.

## Introduction

In hydrostatic drive systems, positive displacement machines are pumps and hydraulic motors. Due to high operating pressures, piston pumps and piston motors dominate in hydrostatic systems^1–5^. Other constructions of positive displacement machines, such as gear^6–10^, gerotor^11^ or vane machines^12^, are also used. Recent years have been a period of intensive development of positive displacement machines, especially hydraulic motors, in which the working mechanism is a special set of non-circular gears. This article is devoted to these machines.

**Figure 1:**
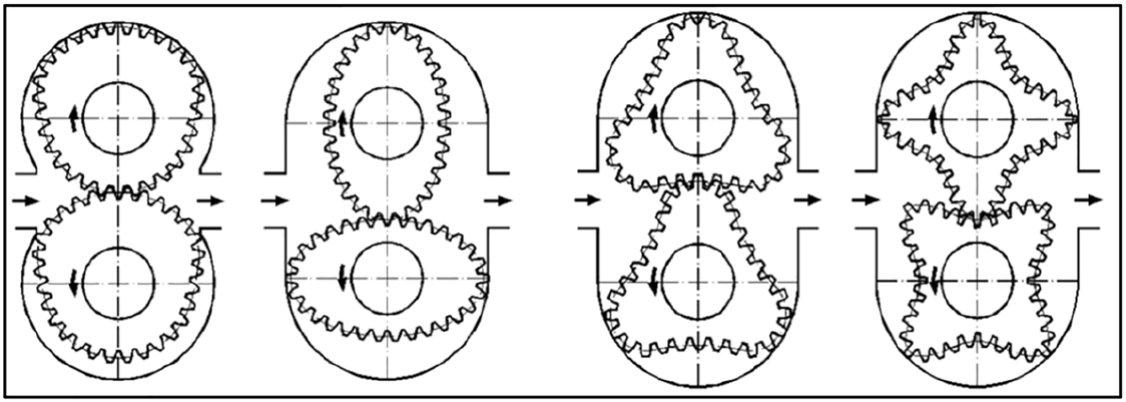
Non-circular gears in positive displacement machine^17^.

The idea of non-circular gears is not new. Non-circular gears were used in many devices to provide irregular motion, which is the transfer (generally) stable input velocity into various output velocities. An example of such devices are clockworks, astronomical devices, electromechanical systems to control and drive non-linear potentiometers, textile machines^13^, mechanical presses^14–16^ and mechanical toys also. Furthermore, from the eighteenth century, the non-circular gears were commonly used in positive displacement machines like in pumps and in flowmeters (Fig. 1)^17^. Both gear transmissions and hydraulic positive displacement machines (Fig. 1) are built with non-circular gears with a constant distance between the axles of these wheels. The methods of designing such gear transmissions are widely described in the literature^13–23^.

**Figure 2:**
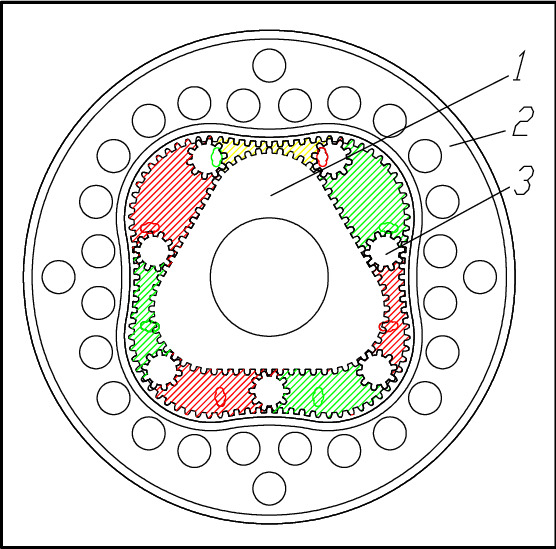
The working mechanism of the first satellite motor (type 3 × 4): 1—rotor, 2—curvature, 3—satellite^24–26^.

While at the end of the nineteenth century the first hydraulic motor with non-circular gears was built^24–26^. This motor was called satellite motor (Fig. 2).

The conception of satellite motor working mechanism is based on mutual cooperation of the external toothed non-circular wheel (called rotor) with the internal toothed non-circular wheel (called curvature) through the round gears (called satellites) between them. The satellites play the role of movable, inter-chamber partitions. Simultaneously, the satellite functions as inflow and outflow dividers when the working chamber passes from the filling phase to the extrusion phase^26^.

By the type of the satellite mechanism should be understood its characteristic feature, which is the number n_R_ of humps on the rotor and the number n_E_ of humps on the curvature. Thus, the type of mechanism will be marked as “n_R_ x n_E_”.

**Figure 3:**
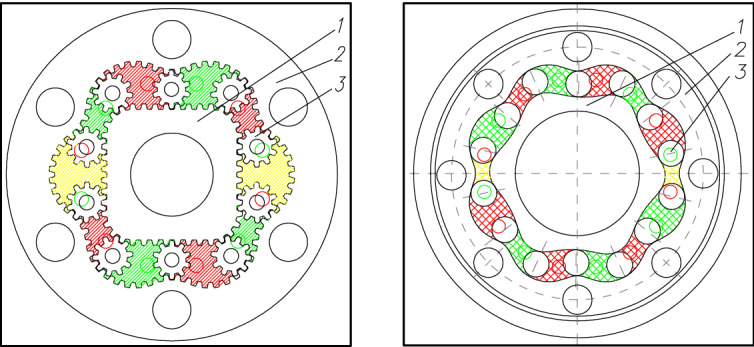
Satellite mechanisms: type 4 × 6 (left) and type 6 × 8 (right): 1—rotor, 2—curvature, 3—satellite^26,28–34^.

**Figure 4:**
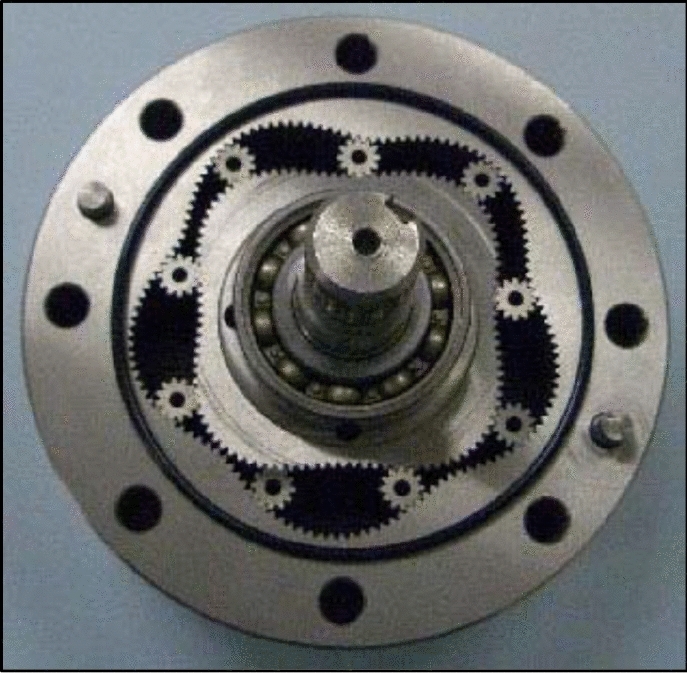
The hydraulic motor with satellite mechanism type 4 × 5. The number of teeth: curvature z_E_ = 130, rotor z_R_ = 104 and satellite z_S_ = 12, tooth module m = 0,5 mm^35,36^.

Currently, hydraulic motors with four types of satellite mechanisms are manufactured (Figs. 2, 3i, 4).

Satellite mechanisms are used not only to build a hydraulic motor, but also to build a pressure intensifier and a pump^36–39^.

According to patents^30,31,34^, the shape of the rotor consists of arcs of circles with different radii and tangents to each other. Similarly, the shape of the curvature is the sum of arcs with different radii. These constructions resulted mainly from the available technologies of their production. Both the teeth of the rotor and the curvature were made by diagonal hobbing with a Fellows tool with the use of special tooling of a slotting machine^40–42^. While the satellites were made using the milling method. Therefore, when designing a satellite mechanism, the available tools (number of chisel teeth, its diameter etc.) should also be taken into account. Moreover, the phenomenon of tooth interference in the mechanism had to be avoided^43^. Therefore, Kujawski in^43^ formed the rotor of the type 4 × 6 mechanism by the arcs of circles connected by straight sections and the construction of curvature consists of only arcs of circles. Kujawski was the first to present the guidelines and basics of designing the satellite mechanisms^43^.

**Figure 5:**
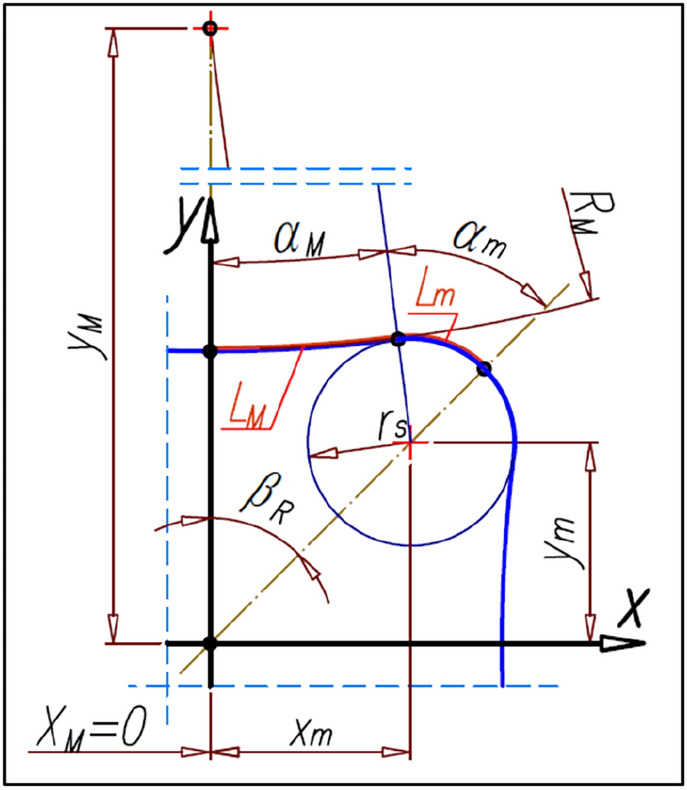
Rotor (four-humped) as a sum of arcs^26^.

In work^26^ a four-humped rotor is composed of arcs tangent to each other are presented (Fig. 5). Dowel Li et al. also developed a methodology of designing the satellite mechanism type 4 × 6 based on circular arc curves of the rotor and curvature^44^.

**Figure 6:**
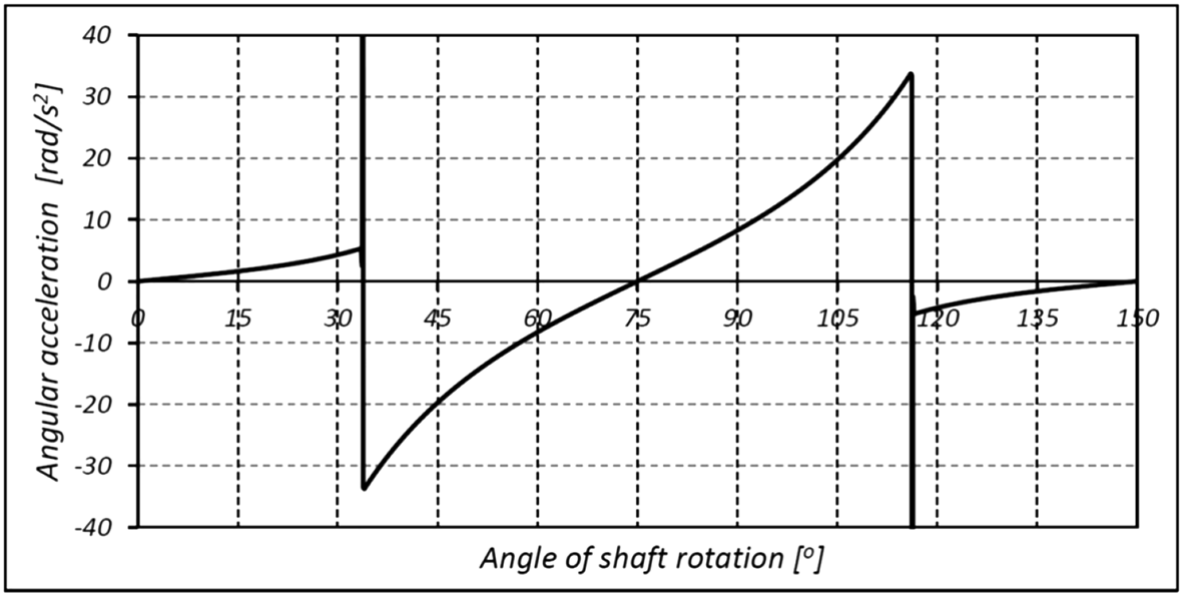
The characteristic of the angular acceleration of satellite in mechanism type 4 × 6 (at the angular speed of rotor ω = 10 rad/s)^26^.

**Figure 7:**
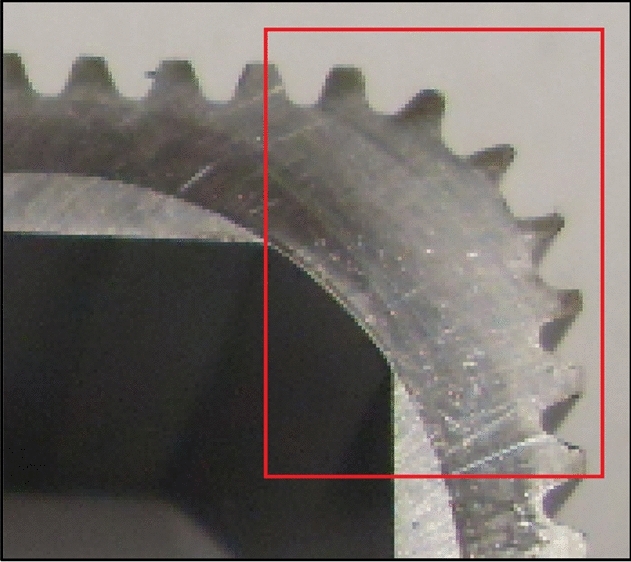
Specific wear of the teeth on the humps of the rotor. Working liquid—emulsion HFA-E. Unknown mechanism operation time^26,45^.

In mechanisms type 3 × 4, 4 × 6 and 6 × 8 the shape of the rotor consists of arcs of circles with different radii and tangents to each other. It was shown in^26^ that in these mechanisms are unfavourably large changes in the satellite’s acceleration at the moment of transition from the convex part of rotor to the concave part, i.e. in point where arcs of circles meet (Fig. 6). The immediate cause of this is a jump change in the radius value at the point of tangency of the circle’s arcs. Thus, in an operating mechanism, especially at high rotational speed, there will be a large mechanical loss which contributes to accelerated wear of the teeth. Figure 7 shows that the wear occurs not only at the points of contact of the arcs but also on the convexity (on the hump) of the rotor. The reason for this is the small radius of this hump^45^.

Currently is possible to manufacture the toothed elements using the wire electrical discharge machining (the so-called WEDM method). This method is already used to manufacture well-known satellite mechanisms, especially the 4 × 6 type, the structures of which were designed to be made by chiseling^44,47,48^. Thus, the WEDM makes it possible to manufacture satellite mechanisms with different rotors and curvature shapes. For example, the rotor of the mechanism type 4 × 5 (Fig. 4) has a circular-sinusoidal shape^35^. The radius r_R_ of the rotor pitch line was described by equation^35^:

1 $${\mathrm{r}}_{\mathrm{R}}={\mathrm{r}}_{\mathrm{Rmin}}+\frac{{\mathrm{r}}_{\mathrm{Rmax}}-{\mathrm{r}}_{\mathrm{Rmin}}}{2}\cdot  \left(1+\mathrm{cos}\left({\mathrm{n}}_{\mathrm{R}}\cdot  {\mathrm{\alpha }}_{\mathrm{R}}+\uppi \right)\right)$$

where r_Rmin_ and r_Rmax_—respectively: minimum and maximum rotor radius, n_R_—the number of the rotor humps, α_R_—angle (Fig. 13).

For the mechanism shown in Fig. 4 is: r_Rmin_ = 22,552 mm and r_Rmax_ = 27.524 mm^35^.

**Figure 8:**
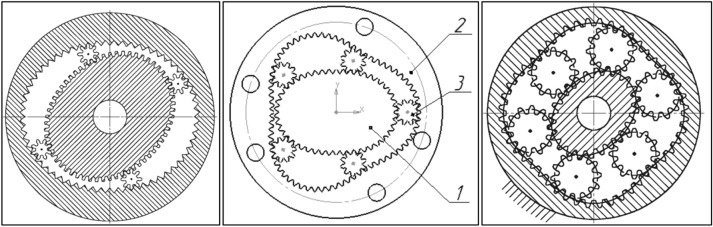
Satellite mechanisms: type 2 × 2 (left), type 2 × 3 (middle) and type 2 × 4 (right), 1—rotor, 2—satellite, 3—curvature^49–56^.

Nowadays are proposed the next concepts of satellite mechanism. Osiecki proposes a satellite mechanism type 2 × 4 with the elliptical shape of the rotor (two humps) and with four humps curvature^45,46^. However, Osiecki did not disclose the methodology of designing curvature and methodology of selection the number of teeth in the mechanism elements (rotor, curvature and satellite). In literature the satellite mechanisms type 2 × 2 and 2 × 3 are also known (Fig. 8)^49–56^.

Volkov at all propose to design satellite mechanisms, like shown in Fig. 8, by specifying, first, the trajectory of the satellite’s center associated with the rotor and with the curvature. In polar coordinates the distance L_SR_ of the satellite center associated with the rotor from the origin of the coordinate system is^49–52^:

2 $${\mathrm{L}}_{\mathrm{SR}}={\mathrm{L}}_{\mathrm{c}}\cdot  \left(1+{\mathrm{k}}_{t}\cdot  \mathrm{f}\left({\mathrm{n}}_{\mathrm{R}}\cdot  {\mathrm{\alpha }}_{\mathrm{SR}}\right)\right)$$

and the distance L_SE_ of the satellite center associated with the curvature from the origin of the coordinate system is^49–52^:

3 $${\mathrm{L}}_{\mathrm{SE}}={\mathrm{L}}_{\mathrm{c}}\cdot  \left(1+{\mathrm{k}}_{t}\cdot  \mathrm{f}\left({\mathrm{n}}_{\mathrm{E}}\cdot  {\mathrm{\alpha }}_{\mathrm{SE}}\right)\right)$$

where f(…)—cyclic function, k_t_—the coefficient that characterizes the non-circularity of the trajectories, L_C_—the radius of the circle to which both trajectories degenerate when k = 0, α_SR_ and α_SE_—polar angles associated with the rotor and curvature respectively.

Next, the rotor and curvature curves are calculated as equidistance’s of the above-mentioned trajectories (2) and (3), assuming the satellite diameter and the number of teeth on the satellite, rotor and curvature^49–52^.

Zhang et al.^57^ and Wang et al.^39^ propose the new satellite mechanism type 4 × 6 with the high-order ellipse shape of the rotor. The pitch curve of the rotor is described in polar coordinates as:

4 $${\mathrm{r}}_{\mathrm{R}}=\frac{A\cdot  \left(1-{k}^{2}\right)}{1+{\mathrm{k}}_{e}\cdot  \mathrm{cos}\left({n}_{R}\cdot  {\mathrm{\alpha }}_{\mathrm{R}}-\pi \right)}$$

where r_R_—the distance between the origin of the coordinate system and a point on the rotor pitch curve, k_e_—the eccentricity of the ellipse, A—the long-axis radius of the ellipse, α_R_—the polar angle of the rotor pitch curve.

Zhang at all are also propose to describe the rotor pitch line as^57^:

5 $${\mathrm{r}}_{\mathrm{R}}={\mathrm{A}}_{h}\cdot  \mathrm{cos}\left({n}_{R}\cdot  {\mathrm{\alpha }}_{\mathrm{R}}\right)+B\cdot  \mathrm{cos}\left(2\cdot  {n}_{R}\cdot  {\mathrm{\alpha }}_{\mathrm{R}}\right)+{\mathrm{r}}_{\mathrm{c}}$$

where r_c_—the radius of basic circle, A_h_—the amplitude of the cousine function, B—coefficient.

According to Zhang, Wang at all the equation describing the curvature pitch curve in polar coordinates is^39,57^:

6 $${\mathrm{r}}_{\mathrm{E}}={\mathrm{r}}_{\mathrm{R}}+2\cdot  \frac{{\mathrm{r}}_{\mathrm{R}}\cdot  {\mathrm{r}}_{\mathrm{S}}}{{\left[{{\mathrm{r}}_{\mathrm{R}}}^{2}+{\left(\frac{d{\mathrm{r}}_{\mathrm{R}}}{d{\mathrm{\alpha }}_{\mathrm{R}}}\right)}^{2}\right]}^{\mathrm{0,5}}}$$

7 $${\mathrm{\alpha }}_{\mathrm{E}}={\int }_{0}^{{\mathrm{\alpha }}_{\mathrm{R}}}\left[{\mathrm{r}}_{\mathrm{R}}+2\cdot  \frac{{\mathrm{r}}_{\mathrm{R}}\cdot  {\mathrm{r}}_{\mathrm{S}}}{{\mathrm{r}}_{\mathrm{E}}}\cdot  \frac{{\left(\frac{d{\mathrm{r}}_{\mathrm{R}}}{d{\mathrm{\alpha }}_{\mathrm{R}}}\right)}^{2}-{\mathrm{r}}_{\mathrm{R}}\cdot  \frac{{d}^{2}{\mathrm{r}}_{\mathrm{R}}}{d{{\mathrm{\alpha }}_{\mathrm{R}}}^{2}}}{{\left[{{\mathrm{r}}_{\mathrm{R}}}^{2}+{\left(\frac{d{\mathrm{r}}_{\mathrm{R}}}{d{\mathrm{\alpha }}_{\mathrm{R}}}\right)}^{2}\right]}^{\mathrm{1,5}}}\right]d{\mathrm{\alpha }}_{\mathrm{R}}$$

where r_E_—the distance between the origin of the coordinate system and a point on the curvature pitch curve, r_S_—radius of the satellite pitch curve, α_E_—the polar angle of the curvature pitch curve.

**Figure 9:**
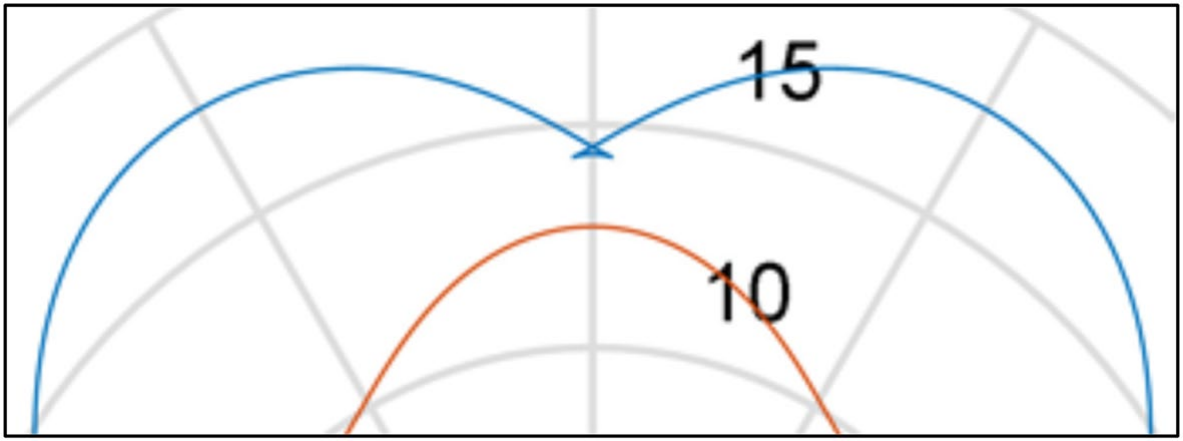
The phenomenon of self-interlacing of the curvature pitch line^57^.

**Figure 10:**
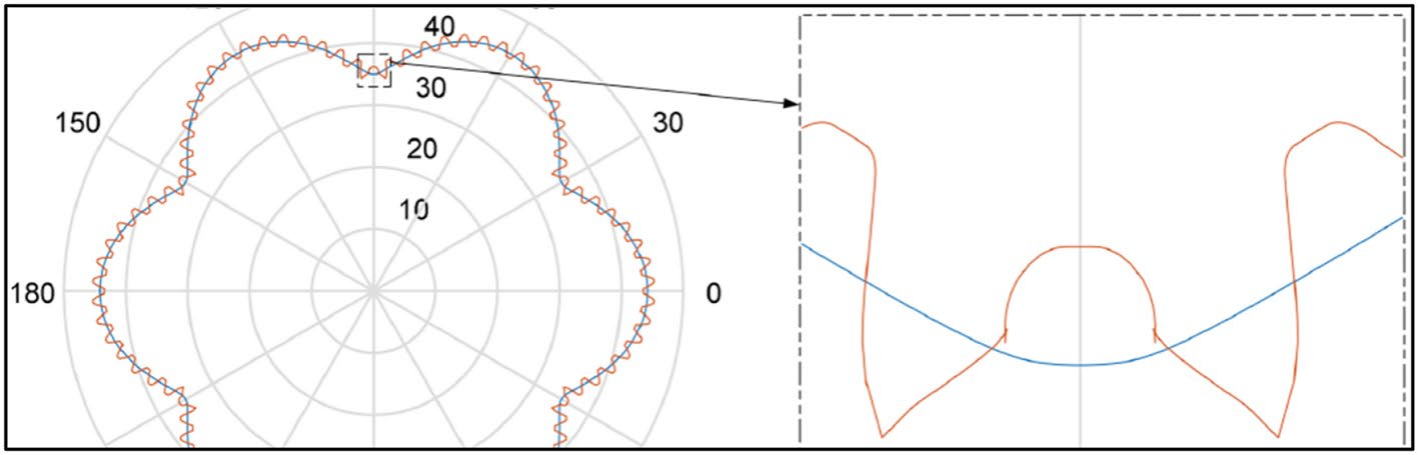
The phenomenon of undercutting the curvature teeth^57^.

Zhang at all indicate that with an inapproprioprate selection of parameters, the curvature is characterized by self-interlacing of the pitch line (Fig. 9) or by undercutting the teeth (Fig. 10).

Furthermore, Zhang at all indicate, when selecting the pitch curve parameters, it is necessary to draw the tooth profile of each gear at the same time to judge its feasibility^57^. It is a certain inconvenience. The next problem to solve is determining how to select the coefficients A_h_ and B in Eq. (5) in solving the undercutting problem of the curvature so that the number of satellite teeth z_S_ is small enough^57^.

In the above-characterized known methods of designing a satellite mechanism, some disadvantages related to the selection of the parameters of the rotor and curvature pitch lines as well as the selection of the number of teeth and their module can also be seen. Therefore, new two methods for designing any type of satellite mechanism for n_E_ > n_R_ are proposed below. The first method allows determining parameters of satellite mechanism for the perfect solution and the second method allows for the correction of the teeth.

## Proposed method of designing a satellite mechanism

In the method of designing a satellite mechanism it is assumed that the satellite plays the role of a chisel. That is in a computer program the satellite chisels the gears of the rotor and curvature. Therefore, the mathematical relationships describing the position of the satellite center in the X–Y coordinate system and the corresponding angle of satellite rotation are also presented below.

### Basic conditions

**Figure 11:**
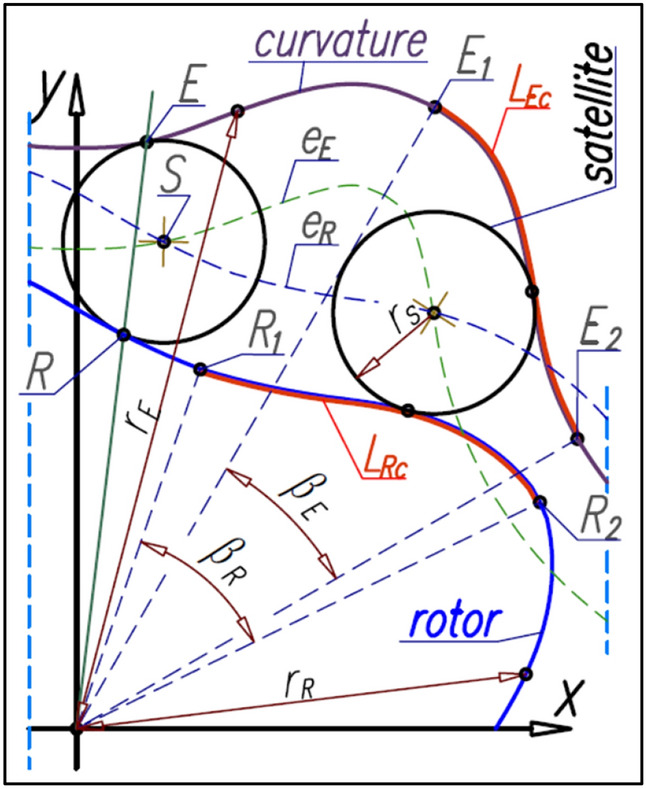
The basic geometrical relationships in satellite mechanism.
- the point R of contact of the satellite with the rotor and the point E of contact of the satellite with the curvature lie on a straight line that passes through the center of rotation of the rotor and curvature (Fig. 11);
- if the curves of the rotor are between points R_1_ and R_2_ and the curves of curvature are between points E_1_ and E_2_ (Fig. 11) and also the tangents to these curves are perpendicular to the leading radii r_R_ (rotor) and r_E_ (curvature) then the length L_Rc_ of the basic curve of the rotor is equal to the length L_Ec_ of the basic curve of curvature:
  8 $${\mathrm{L}}_{\mathrm{Rc}}={\mathrm{L}}_{\mathrm{Ec}}$$
- the central angle β_R_ that covers one half of the cycle of the rotor pitch curve (corresponding to length L_Rc_) is:
  9 $${\upbeta }_{\mathrm{R}}=\frac{\uppi }{{\mathrm{n}}_{\mathrm{R}}}$$
  where n_R_ is the number of the rotor humps;
- the central angle β_E_ that covers one half of the cycle of the curvature’s pitch curve (corresponding to length L_Ec_) is:
  10 $${\upbeta }_{\mathrm{E}}=\frac{\uppi }{{\mathrm{n}}_{\mathrm{E}}}$$
  where n_E_ is the number of the curvature humps;
- the number of humps of the curvature is greater than the number of humps of the rotor (n_E_ > n_R_).

It is assumed that for each radius r_s_ of the satellite’s pitch circle and for each rotor pitch line exists a corresponding to them a pitch line of curvature, which fulfils the conditions of perfect cooperation. These conditions are as follows^43^:

- in each mutual position of rotor and curvature, obtained as a result of the rotation of one of them around the other, the pitch circles of all satellites must be tangent to the pitch lines of the rotor and the curvature;
- the pitch circles of all satellites must pitch without slipping on the pitch lines of the rotor and the curvature;
- on all length of rotor’s pitch line and on all length of the curvature’s pitch line must exist such humps that make the possible pitch of the satellite’s pitch circle on the external side of the rotor’s pitch line and on the internal side of the curvature’s pitch line;
- the rotors and curvature’s pitch lines should be cyclically changing curves and must not overlap with mutual rotational displacement;
- the centers S of the satellites should be located at the intersection point of the equidistant e_R_ of the pitch line of the rotor with the equidistant e_E_ of the pitch line of the curvature (equidistance are the tracks of the satellite centers, which arise as a result of the pitch the satellite on the pitch lines of the rotor and the curvature) (Fig. 11);

### Proposed sequence of proceeding during the design

When starting the design of the satellite mechanism, the first step is to take the number of humps n_R_ on the rotor and the number of humps n_E_ on the curvature. Next the radius r_c_ of the rotor basic circle must be chosen.

**Figure 12:**
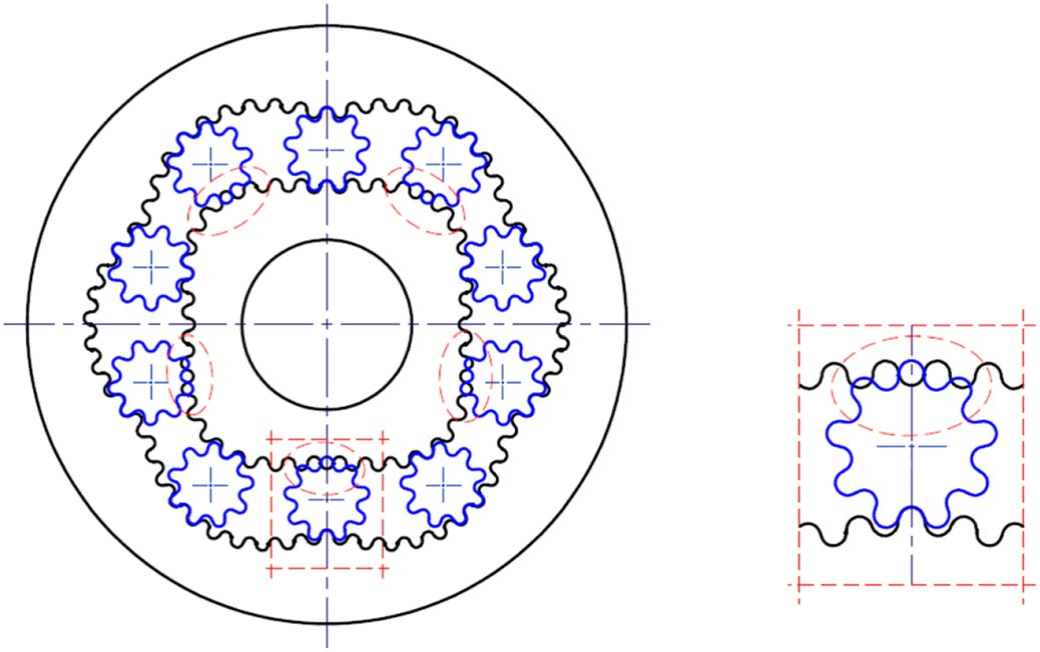
Satellite mechanism type 4 × 6 with incorrectly selected parameters: z_S_ = 9, z_Rc_ = 4.75, z_R_ = 38, z_E_ = 57. Every second satellite cannot be inserted into the mechanism.

When designing a satellite mechanism it should be remembered that:

- the number of teeth z_Rc_ in the range of rotor angle β_R_ (Fig. 16) is:
  11 $${\mathrm{z}}_{\mathrm{Rc}}=\frac{{\mathrm{L}}_{\mathrm{Rc}}}{\uppi \cdot  \mathrm{m}}$$
- because the condition (8) must be met, then:
  12 $${\mathrm{z}}_{\mathrm{Ec}}={\mathrm{z}}_{\mathrm{Rc}}$$
- the number of teeth z_R_ on the rotor is:
  13 $${\mathrm{z}}_{\mathrm{R}}=2\cdot  {\mathrm{n}}_{\mathrm{R}}\cdot  {\mathrm{z}}_{\mathrm{Rc}}$$
- the number of teeth z_E_ on the curvature is:
  14 $${\mathrm{z}}_{\mathrm{E}}=2\cdot  {\mathrm{n}}_{\mathrm{E}}\cdot  {\mathrm{z}}_{\mathrm{Rc}}$$
- numbers of teeth z_R_ and z_E_ must be integer;
- the number of teeth 2z_Rc_ on the rotor hump (and the same on the curvature hump) must be an integer. Otherwise, despite the fulfilment of the condition (8) and the total number of rotor teeth z_R_ and curvature teeth z_E_, the satellite mechanism cannot be assembled correctly. An example of such mechanism is presented in Fig. 12.

#### The first method: searching for the perfect solution

Having the number of humps n_R_ and n_E_ should be sequentially:

1. to adopt radius r_c_ of rotor basic circle;
2. using the iteration method should be searched the amplitude A_h_ and the satellite radius r_S_ until the condition (8) is met with simultaneous fulfilment of the condition (54);
3. using the iteration method should be searched the number of satellite teeth z_S_ so that the numbers of teeth z_R_ and z_E_ are integer;
4. to calculate teeth module m according to the following formula:
  15 $$\mathrm{m}=2\cdot  \frac{{\mathrm{r}}_{\mathrm{S}}}{{\mathrm{z}}_{\mathrm{S}}}$$
  The calculated value of module m does have to correspond to the normalized value;
5. to adopt the desired normalized value of module m_st_;
6. all the parameters of the satellite mechanism should be scaled by a value:
  16 $$\mathrm{i}=\frac{\mathrm{m}}{{\mathrm{m}}_{\mathrm{St}}}$$

The search for the parameters of satellite mechanism, ie A_h_, r_S_ and z_S_ so that the condition (54) was met may prove impossible. Then it is worth to allowing a certain difference δ of the lengths L_Rc_ and L_Ec_, but not greater than the limit value δ_b_, that is:

17 $$\left|{\mathrm{L}}_{\mathrm{Rc}}-{\mathrm{L}}_{\mathrm{Ec}}\right|=\updelta \le {\updelta }_{\mathrm{b}}$$

The value δ_b_ is justified, for example, by the fact that depending on the processing technology, different accuracy of the manufacturing of satellite mechanism elements is obtained.

#### The second method

Having the number of humps n_R_ and n_E_ should be sequentially:

1. to adopt the basic circle radius r_c_ of the rotor;
2. to adopt the amplitude A_h_;
3. by iteration method to search the satellite radius r_S_ until the condition (8) is met while meeting the condition (54) (presented in “Method 2”);
4. to adopt the number of teeth z_Rc_ (keeping in mind that the number of teeth 2z_Rc_ on the rotor hump should be integer);
5. to calculate module m by transforming the formula (11);
6. to calculate the number of teeth z_S_ by transforming the formula (15). The obtained value z_S_ need not be an integer;
7. if the calculated value of z_S_ is not integer then the total number of the satellite teeth z_Sst_ should be adopted. It is recommended that:
  18 $$\left|{\mathrm{z}}_{\mathrm{S}}-{\mathrm{z}}_{\mathrm{Sst}}\right|=\mathrm{min}$$
  19 $${\mathrm{z}}_{\mathrm{Sst}}<{\mathrm{z}}_{\mathrm{S}}$$
8. to apply the P-O correction of the teeth. The minimum value of the correction coefficient is:
  20 $${\mathrm{x}}_{\mathrm{smin}}=\frac{{\mathrm{z}}_{\mathrm{S}}-{\mathrm{z}}_{\mathrm{Sst}}}{2}$$
9. in order to generate corrected teeth of the rotor and the curvature, the angle γ_Sst_ of satellite rotation relative to its center S should be calculated according to the following formula:
  21 $${\upgamma }_{\mathrm{Sst}}=\frac{{\mathrm{L}}_{\mathrm{R}}}{{\mathrm{r}}_{\mathrm{Sst}}}+\mathrm{arctan}\left(\frac{{\mathrm{x}}_{\mathrm{R}}-{\mathrm{x}}_{\mathrm{S}}}{{\mathrm{y}}_{\mathrm{R}}-{\mathrm{y}}_{\mathrm{S}}}\right)$$
  where:
  22 $${\mathrm{r}}_{\mathrm{Sst}}=\mathrm{m}\cdot  \frac{{\mathrm{z}}_{\mathrm{Sst}}}{2}$$

### Rotor designing: the coordinates of the rotor pitch line

**Figure 13:**
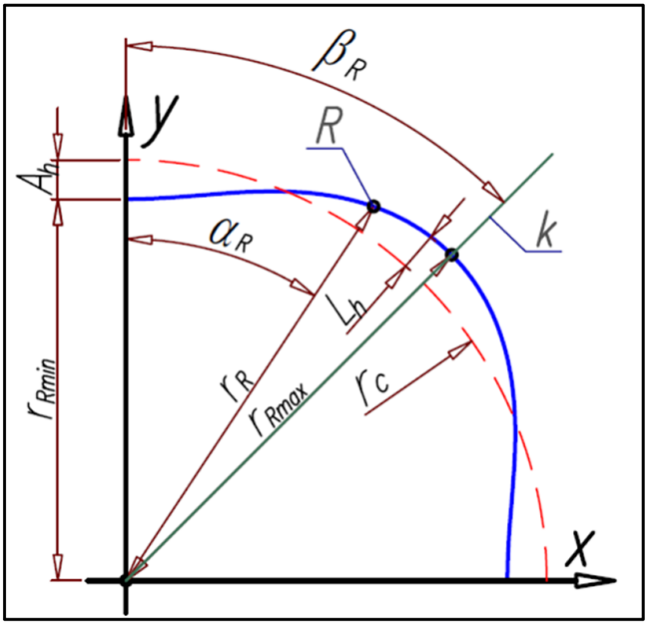
The basic parameters of the rotor.

According to the basic conditions shown in “Basic conditions” the rotor’s pitch lines should be a cyclically changing curve. Furthermore, the rotor should have a total number of humps n_R_ evenly distributed over the entire circumference of the rotor. Therefore, the radius r_R_ of the rotor pitch line can be defined by any cyclical function type r_R_ = f(α_R_), for example functions (1), (4), (5) and any others. A schematic sketch of a quarter rotor with the basic geometrical dimensions is shown in Fig. 13.

The coordinates (x_R_,y_R_) of the point R on the rotor pitch line are:

23 $${\mathrm{x}}_{\mathrm{R}}={\mathrm{r}}_{\mathrm{R}}\cdot  \mathrm{sin}{\mathrm{\alpha }}_{\mathrm{R}}$$

24 $${\mathrm{y}}_{\mathrm{R}}={\mathrm{r}}_{\mathrm{R}}\cdot  \mathrm{cos}{\mathrm{\alpha }}_{\mathrm{R}}$$

The most distant point of the rotor pitch line from the center of rotation of this rotor (i.e. from the origin of the coordinate system) designates the straight-line k which is the axis of symmetry of the rotor hump (Fig. 13). If α_S_ = α_R_ = β_R_ then the satellite center S and the tangent point R of the satellite with the rotor lie on the straight-line k (Fig. 16b). The angle β_R_ can be calculated from the formula (10).

### The coordinates of the satellite center

**Figure 14:**
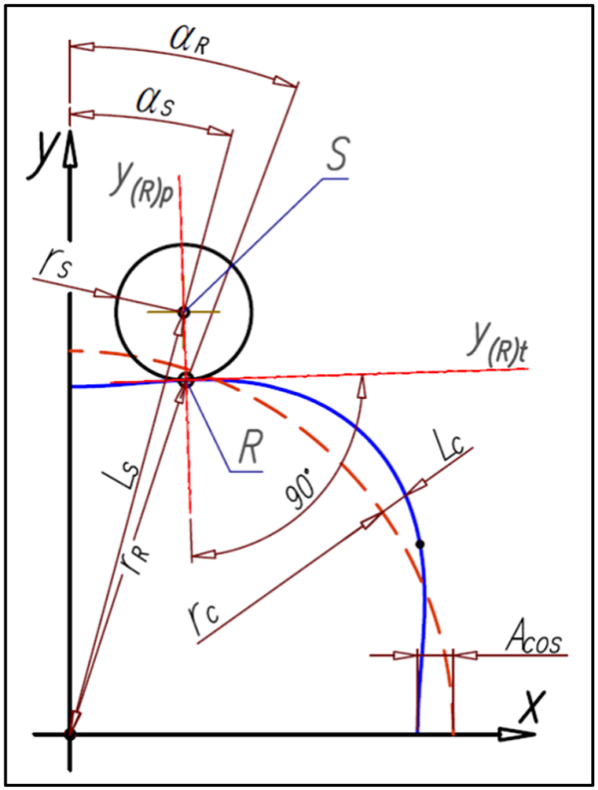
The coordinates of the satellite center.

The satellite pitch circle with radius r_s_ must be tangent in the point R to the rotor pitch line (Fig. 14).

The coordinates (x_S_,y_S_) of the satellite center S can be calculated according to the following formulas:

25 $${\mathrm{x}}_{\mathrm{S}}={\mathrm{x}}_{\mathrm{R}}\pm {\mathrm{r}}_{\mathrm{S}}\cdot  \frac{1}{\sqrt{{1+{\mathrm{a}}_{\left(\mathrm{R}\right)\mathrm{p}}}^{2}}}$$

26 $${\mathrm{y}}_{\mathrm{S}}={\mathrm{y}}_{\mathrm{R}}\pm {\mathrm{r}}_{\mathrm{S}}\cdot  \frac{{\mathrm{a}}_{\left(\mathrm{R}\right)\mathrm{p}}}{\sqrt{{1+{\mathrm{a}}_{\left(\mathrm{R}\right)\mathrm{p}}}^{2}}}$$

where a_(R)p_ is the slope of the straight-line y_(R)p_ perpendicular to the tangent line y_(R)t_ in the point R (Fig. 14). If a_(R)p_ < 0 then in formulas (25) and (26) is a “−” sign instead of the “±” sign. But for a_(R)p_ ≥ 0 is sign “+”.

The angular position of the satellite center S (angle α_S_ in Fig. 14) about the axis OY can be calculated from the following formula:

27 $${\mathrm{\alpha }}_{\mathrm{S}}=\mathrm{arctan}\left(\frac{{\mathrm{x}}_{\mathrm{S}}}{{\mathrm{y}}_{\mathrm{S}}}\right)$$

whereas the distance L_S_ of satellite center S from the origin of the coordinate system is:

28 $${\mathrm{L}}_{\mathrm{S}}=\sqrt{{\mathrm{x}}_{\mathrm{S}}^{2}+{\mathrm{y}}_{\mathrm{S}}^{2}}$$

### The angle of satellite rotation

**Figure 15:**
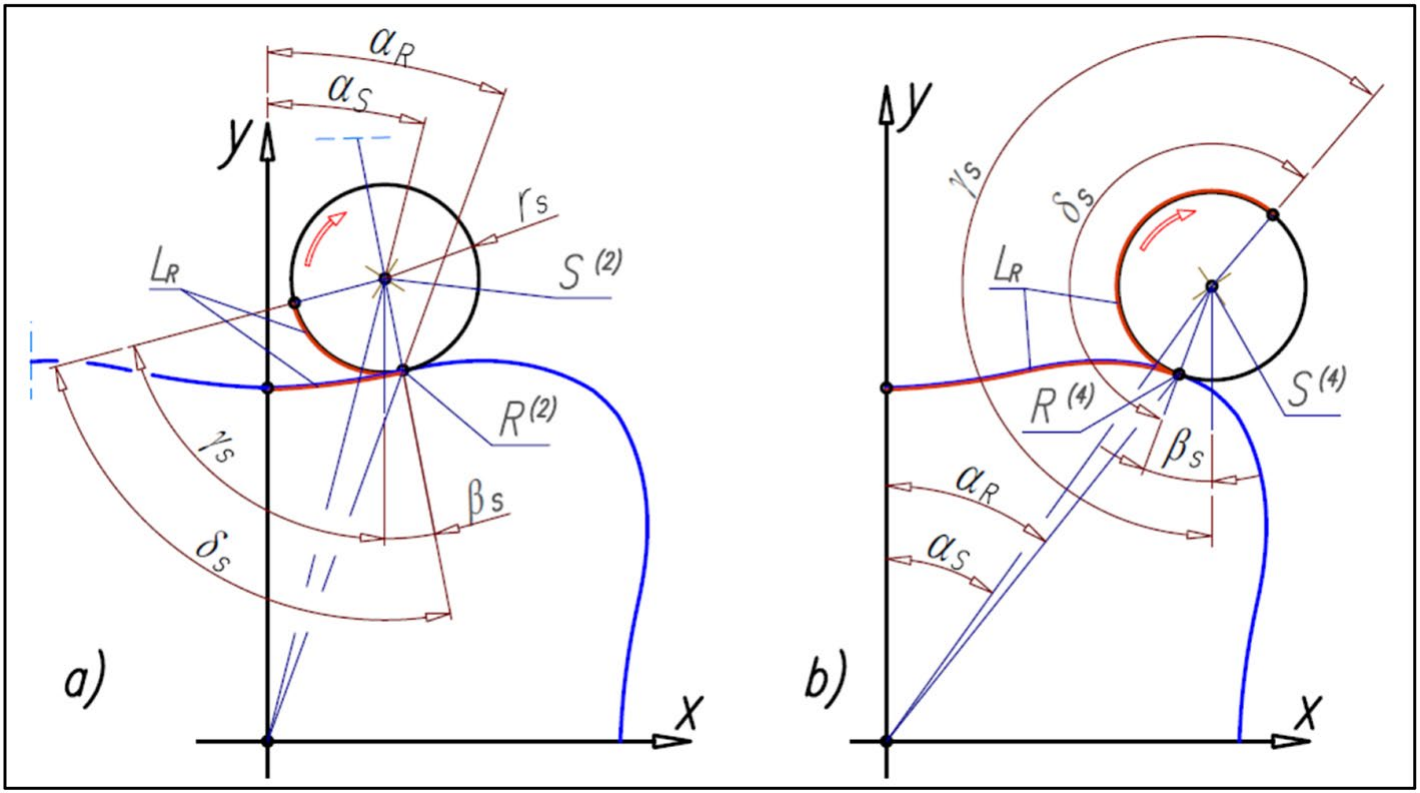
Angles of the satellite.

Each position of satellite center S described by formulas (25) and (26) is assigned the angle γ_S_ of the satellite rotation around the center S (Fig. 15).

The angle γ_S_ of satellite rotation around its center S can be calculated according to the following formula:

29 $${\upgamma }_{\mathrm{S}}=\frac{{\mathrm{L}}_{\mathrm{R}}}{{\mathrm{r}}_{\mathrm{S}}}+\mathrm{arctan}\left(\frac{{\mathrm{x}}_{\mathrm{R}}-{\mathrm{x}}_{\mathrm{S}}}{{\mathrm{y}}_{\mathrm{R}}-{\mathrm{y}}_{\mathrm{S}}}\right)$$

### Curvature designing

**Figure 16:**
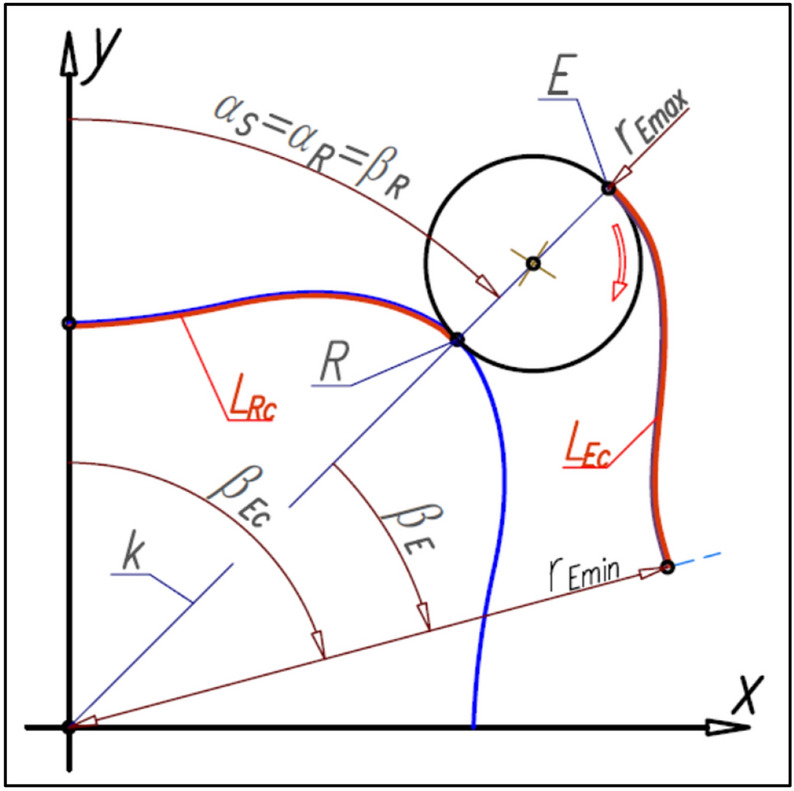
Characteristics angles of the rotor and the curvature and the tangency points of the satellite with the rotor and curvature.

The number n_E_ of the curvature humps must be greater than the number n_R_ of the rotor humps (n_E_ > n_R_). If the satellite center S and the tangency point R of the satellite with the rotor lie on the straight-line k then this line is the symmetry axis of the curvature humps. The tangency point E of the satellite with the curvature lies on the line k also (Fig. 16). Furthermore for r_R_ = r_Rmin_ is r_E_ = r_Emin_ and for r_R_ = r_Rmax_ is r_E_ = r_Emax_.

The number n_E_ of curvature humps corresponds to the angle β_E_, which can be calculated from the formula (10). Formulas (9) and (10) follow that:

30 $${\upbeta }_{\mathrm{R}}\cdot  {\mathrm{n}}_{\mathrm{R}}={\upbeta }_{\mathrm{E}}\cdot  {\mathrm{n}}_{\mathrm{E}}$$

and hence:

31 $${\upbeta }_{\mathrm{E}}=\frac{{\mathrm{n}}_{\mathrm{R}}}{{\mathrm{n}}_{\mathrm{E}}}\cdot  {\upbeta }_{\mathrm{R}}$$

Furthermore, if the satellite moves in relation to the rotor by the angle β_R_ then the curvature will rotate by the angle β_Ec_ (Fig. 16):

32 $${\upbeta }_{\mathrm{Ec}}={\upbeta }_{\mathrm{R}}+{\upbeta }_{\mathrm{E}}={\upbeta }_{\mathrm{R}}\cdot  \left(1+\frac{{\mathrm{n}}_{\mathrm{R}}}{{\mathrm{n}}_{\mathrm{E}}}\right)=\uppi \cdot  \left(\frac{1}{{\mathrm{n}}_{\mathrm{R}}}+\frac{1}{{\mathrm{n}}_{\mathrm{E}}}\right)$$

**Figure 17:**
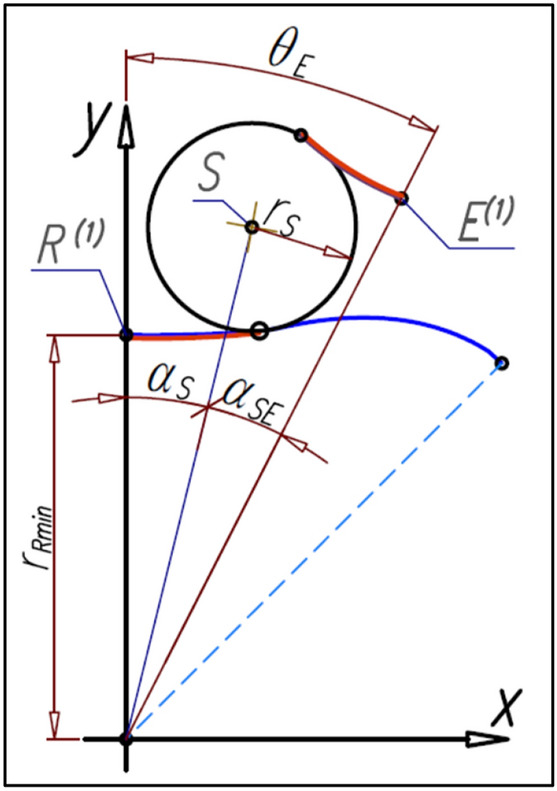
Relations between angular position α_S_ of satellite and the angle θ_E_ of curvature rotate.

If the relations (31) and (32) exist between the angles β_R_, β_E_ and β_Ec_ then the following relations between the angles α_S_, α_SE_ and θ_E_ are also true (Fig. 17):

33 $${\mathrm{\alpha }}_{\mathrm{SE}}=\frac{{\mathrm{n}}_{\mathrm{R}}}{{\mathrm{n}}_{\mathrm{E}}}\cdot  {\mathrm{\alpha }}_{\mathrm{S}}$$

34 $${\uptheta }_{\mathrm{E}}={\mathrm{\alpha }}_{\mathrm{S}}+{\mathrm{\alpha }}_{\mathrm{SE}}={\mathrm{\alpha }}_{\mathrm{S}}\cdot  \left(1+\frac{{\mathrm{n}}_{\mathrm{R}}}{{\mathrm{n}}_{\mathrm{E}}}\right)$$

In order to determine the curvature pitch line, the set of coordinates (x_E_,y_E_) of the point E should be calculated. These coordinates can be calculated by two methods.

#### Method 1: according to three temporaries centers of rotation

**Figure 18:**
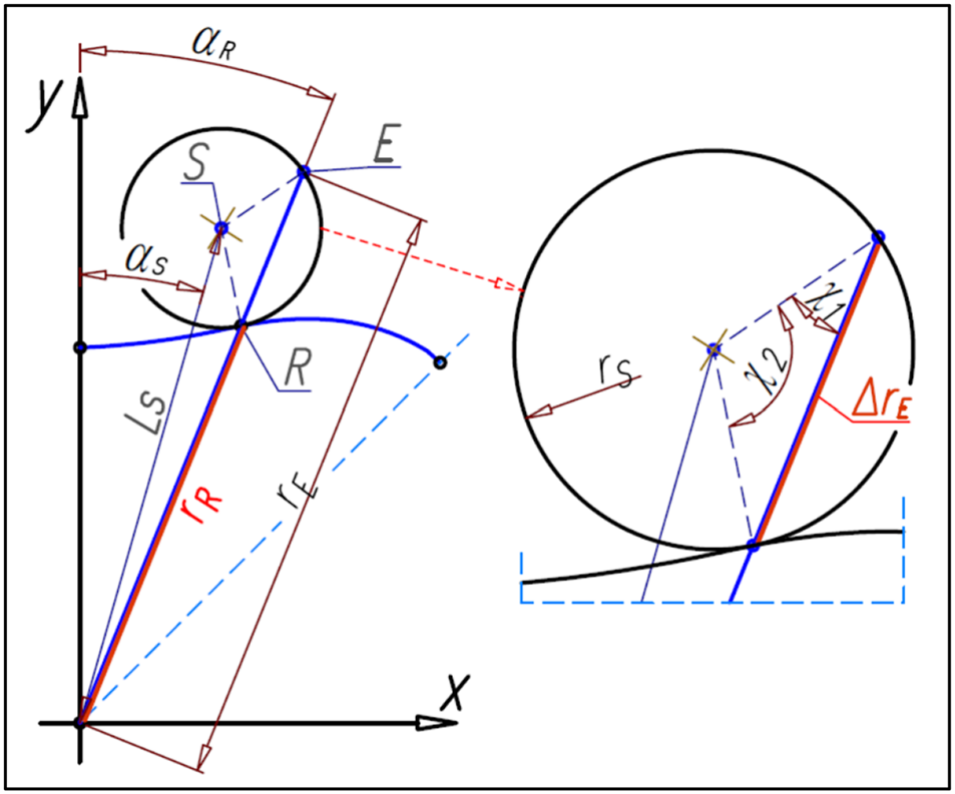
Tangency point E of the satellite with the curvature.

For any position of the satellite the tangency point E of the satellite with the curvature lies on the straight line that passes through the center of rotation of the rotor and the tangency point R of the satellite with the rotor (Fig. 18).

The coordinates of the point E are as follows:

35 $${\mathrm{x}}_{\mathrm{E}}={\mathrm{r}}_{\mathrm{E}}\cdot  \mathrm{sin}\left({\mathrm{\alpha }}_{\mathrm{R}}\right)$$

36 $${\mathrm{y}}_{\mathrm{E}}={\mathrm{r}}_{\mathrm{E}}\cdot  \mathrm{cos}\left({\mathrm{\alpha }}_{\mathrm{R}}\right)$$

where:

37 $${\mathrm{r}}_{\mathrm{E}}={\mathrm{r}}_{\mathrm{R}}+\Delta {r}_{E}$$

38 $$\Delta {r}_{E}={\mathrm{r}}_{S}\cdot  \sqrt{2\cdot  \left(\mathrm{cos}\left(\pi -2\cdot  {\upchi }_{1}\right)\right)}$$

39 $${\upchi }_{1}=arcsin\left(\frac{{L}_{S}}{{r}_{S}}\cdot  \mathrm{sin}\left({\mathrm{\alpha }}_{\mathrm{R}}-{\mathrm{\alpha }}_{\mathrm{S}}\right)\right)$$

**Figure 19:**
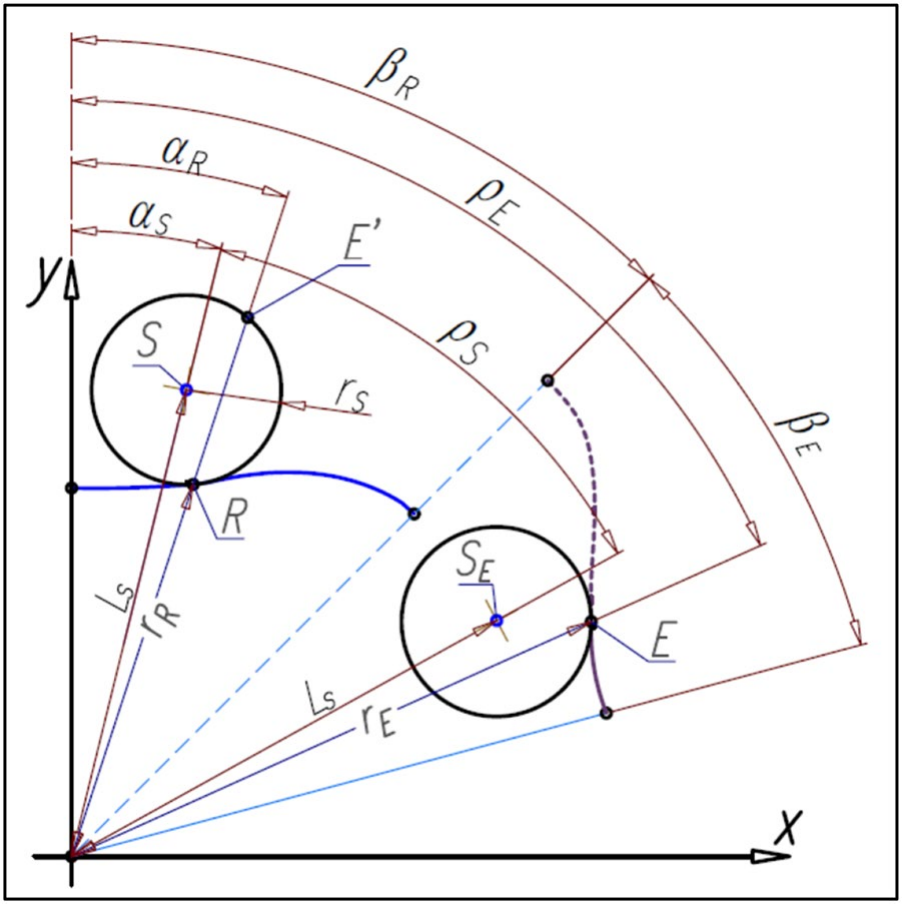
Determining of the curvature pitch line—angular relations.

For any location of the satellite center S relative to the rotor corresponds the location of the center S_E_ of this satellite relative to the curvature (Fig. 19). The satellite pitch circle with center in point S_E_ is tangent the pitch line of the curvature. The angle ρ between points S and S_E_ can be calculated from the following formula:

40 $$\uprho ={\upbeta }_{\mathrm{R}}+{\upbeta }_{\mathrm{E}}-{\mathrm{\alpha }}_{\mathrm{S}}-{\mathrm{\alpha }}_{\mathrm{SE}}=\left(\frac{{\mathrm{n}}_{\mathrm{R}}}{{\mathrm{n}}_{\mathrm{E}}}+1\right)\cdot  \left(\frac{\uppi }{{\mathrm{n}}_{\mathrm{R}}}-{\mathrm{\alpha }}_{\mathrm{S}}\right)$$

whereas the coordinates (x_SE_,y_SE_) of the point S_E_ are:

41 $${\mathrm{x}}_{\mathrm{SE}}={\mathrm{L}}_{\mathrm{S}}\cdot  \mathrm{sin}\left(\uprho +{\mathrm{\alpha }}_{\mathrm{S}}\right)$$

42 $${\mathrm{y}}_{\mathrm{SE}}={\mathrm{L}}_{\mathrm{S}}\cdot  \mathrm{cos}\left(\uprho +{\mathrm{\alpha }}_{\mathrm{S}}\right)$$

where L_S_ is expressed by the formula (28).

The coordinates (x_E_,y_E_) of the point E (Fig. 19) can be calculated from formulas:

43 $${\mathrm{x}}_{\mathrm{E}}={\mathrm{r}}_{\mathrm{E}}\cdot  \mathrm{sin}\left({\uprho }_{E}\right)$$

44 $${\mathrm{y}}_{\mathrm{E}}={\mathrm{r}}_{\mathrm{E}}\cdot  \mathrm{cos}\left({\uprho }_{E}\right)$$

where:

45 $$ {\text{r}}_{{\text{E}}} = \sqrt {{\text{x}}_{{{{{\text{E}}^{\prime}}}}}^{2} + {\text{y}}_{{{{{\text{E}}^{\prime}}}}}^{2} } $$

46 $${\uprho }_{\mathrm{E}}={\uprho }_{S}+{\mathrm{\alpha }}_{\mathrm{R}}$$

47 $${\mathrm{\alpha }}_{\mathrm{R}}=\mathrm{arccos}\left(\frac{{\mathrm{x}}_{\mathrm{R}}}{{\mathrm{r}}_{\mathrm{R}}}\right)$$

#### Method 2

##### Graphical interpretation of determining the curvature pitch line

**Figure 20:**
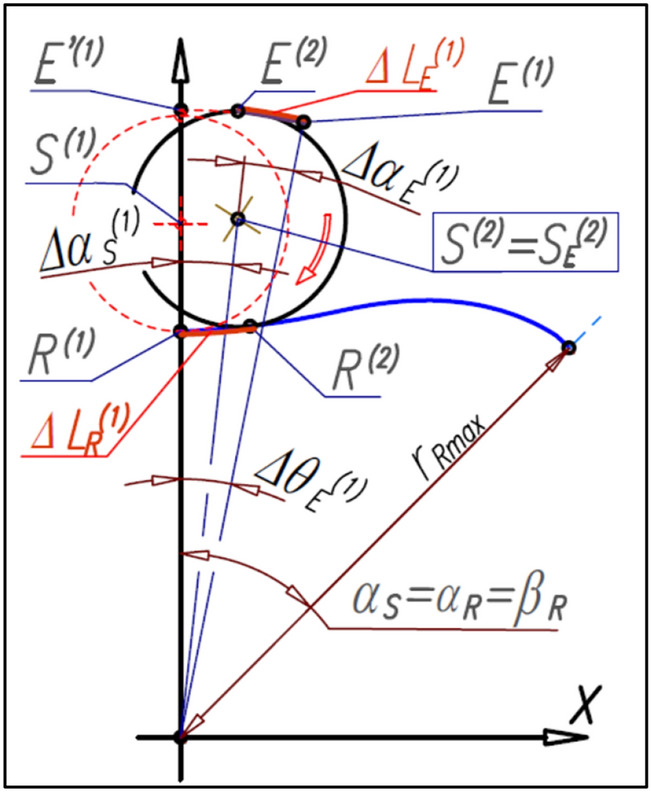
The tangency points E of satellite with the curvature.

**Figure 21:**
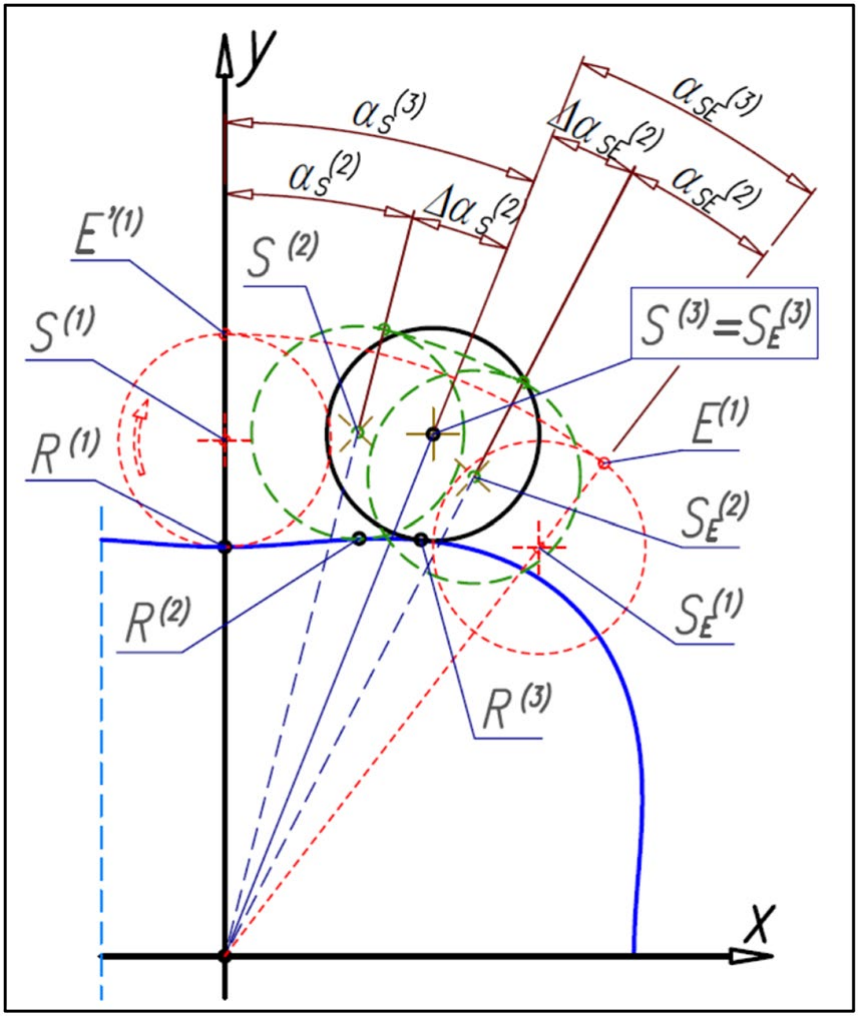
The next positions of the satellite relative to the rotor and the positions of the satellite relative to the curvature—the determination of the shape of the curvature pitch line.

If the satellite is rolled along the rotor pitch line with the elementary angle ∆α_S_^(1)^ then the initial tangency point E’^(1)^ of the satellite with the curvature rotate with the angle ∆α_E_^(1)^ and is in a new position E^(1)^ (Fig. 20). The satellite pitch circle in a new position (point S^(2)^ according to Fig. 20) is tangent to the curvature pitch line. The satellite center is common for satellite position relative to the rotor and satellite position relative to the curvature, that is S^(2)^ = S_E_^(2)^. The next displacement of the satellite with the angle ∆α_S_^(2)^ (Fig. 21) forces the rotation of both the satellite center S^(2)^ and the point E^(1)^ with the angle ∆α_E_^(2)^. Therefore the new position of satellites relative to the curvature are S_E_^(1)^, S_E_^(2)^ and S_E_^(3)^. Wherein, the satellite center position S_E_^(3)^ is the same as the position S^(2)^ relative to the rotor, that is S^(3)^ = S_E_^(3)^. In the next steps the same is done until α_S_ = α_R_ = β_R_.

The displacement of the satellite with the angle ∆α_S_^(1)^ causes the tangency point R will travel the distance ∆L_R_^(1)^ (Fig. 20). The same distance must travel the tangency point E, that is:

48 $${\Delta {\mathrm{L}}_{\mathrm{E}}}^{(1)}={\Delta {\mathrm{L}}_{\mathrm{R}}}^{(1)}$$

After the displacement of the satellite with the angle ∆α_S_^(1)^ the point E^(2)^ is the new tangency point of this satellite with the curvature (Fig. 16). The next displacement of the satellite with the angle ∆α_S_^(2)^ forces the rotation of the points E^(1)^ and E^(2)^ (i.e. the current point and the previous points) with the angle ∆α_E_^(2)^. In the next steps, the same is done until α_S_ = α_R_ = β_R_.

**Figure 22:**
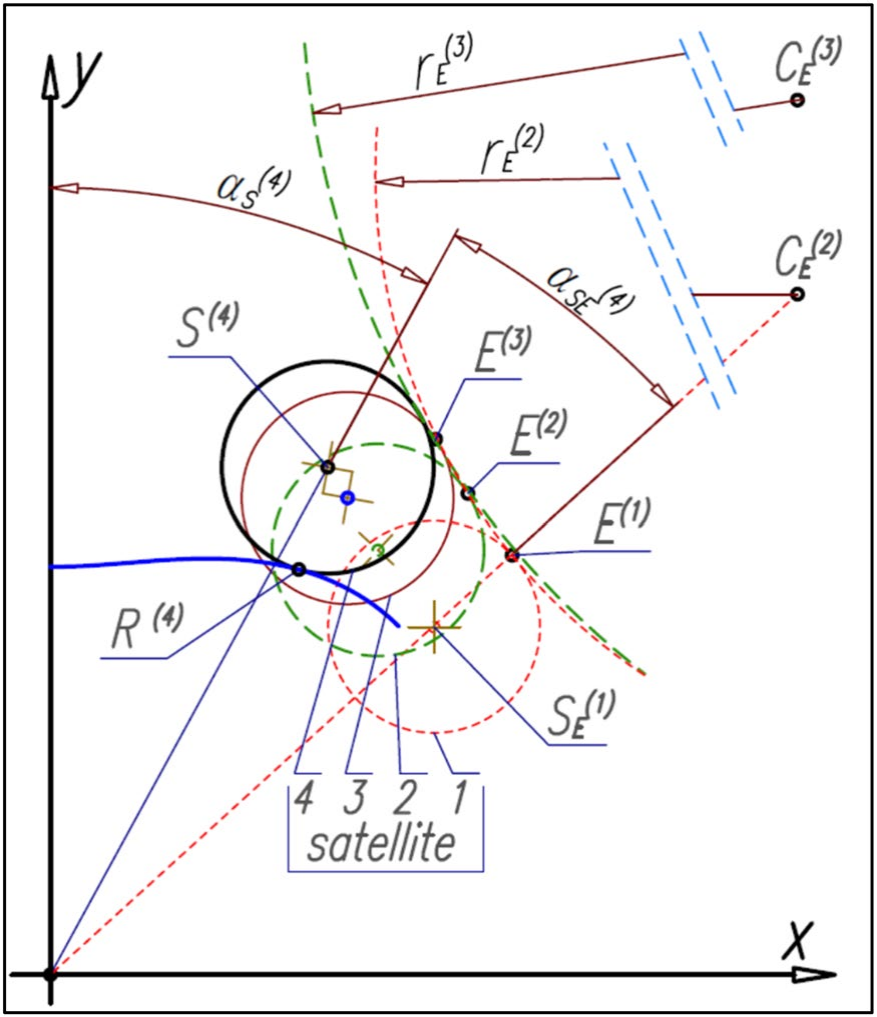
The satellites positions relative to the curvature—the determination of the curvature pitch line.

It is more advantageous to determine the curvature pitch line only after determining the entire set of satellite centers S_E_, i.e. after determining all centers S_E_ with the step of ∆α_E_). This method is illustrated in Fig. 22. The circles with the radius r^E^ and with the center C_E_ are tangent to the three next satellites. That is, the circle with the radius r_E_^(2)^ is tangent to satellites 1, 2 and 3. The point E^(2)^ is the tangency point of the circle with the radius r_E_^(2)^ with the satellite 2. Similarly, the circle with the radius r_E_^(3)^ is tangent to the satellites 2, 3 and 4 and also the point E^(3)^ is the tangency point of the circle with the radius r_E_^(3)^ with the satellite 3.

##### Analytical method of determinig curvature pitch line

The coordinates (x_E_^(1)^,y_E_^(1)^) of the first point E^(1)^ of the curvature (Fig. 22) should be calculated from formulas (43) and (44). But coordinates (x_E_^(i)^,y_E_^(i)^) of the next points E^(i)^ of the curvature should be calculated according to the method shown in Fig. 22. That is, the coordinates (x_CE_^(i)^,y_CE_^(i)^) of the center of the circle with the radius r_E_^(i)^ (with the number (i)) can be calculated from formulas:

49 $${\mathrm{x}}_{\mathrm{CE}}^{(\mathrm{i})}=\frac{1}{2}\cdot  \frac{{\mathrm{y}}_{\mathrm{SE}}^{(\mathrm{i}-1)}-{\mathrm{y}}_{\mathrm{SE}}^{(\mathrm{i}+1)}+\frac{{\left({\mathrm{x}}_{\mathrm{SE}}^{(\mathrm{i})}\right)}^{2}-{\left({\mathrm{x}}_{\mathrm{SE}}^{(\mathrm{i}-1)}\right)}^{2}}{{\mathrm{y}}_{\mathrm{SE}}^{(\mathrm{i})}-{\mathrm{y}}_{\mathrm{SE}}^{(\mathrm{i}-1)}}-\frac{{\left({\mathrm{x}}_{\mathrm{SE}}^{(\mathrm{i}+1)}\right)}^{2}-{\left({\mathrm{x}}_{\mathrm{SE}}^{(\mathrm{i})}\right)}^{2}}{{\mathrm{y}}_{\mathrm{SE}}^{(\mathrm{i}+1)}-{\mathrm{y}}_{\mathrm{SE}}^{(\mathrm{i})}}}{\frac{{\mathrm{x}}_{\mathrm{SE}}^{(\mathrm{i})}-{\mathrm{x}}_{\mathrm{SE}}^{(\mathrm{i}+1)}}{{\mathrm{y}}_{\mathrm{SE}}^{(\mathrm{i}+1)}-{\mathrm{y}}_{\mathrm{SE}}^{(\mathrm{i})}}+\frac{{\mathrm{x}}_{\mathrm{SE}}^{(\mathrm{i})}-{\mathrm{x}}_{\mathrm{SE}}^{(\mathrm{i}-1)}}{{\mathrm{y}}_{\mathrm{SE}}^{(\mathrm{i})}-{\mathrm{y}}_{\mathrm{SE}}^{(\mathrm{i}-1)}}}$$

50 $${\mathrm{y}}_{\mathrm{CE}}^{(\mathrm{i})}=\frac{{\mathrm{x}}_{\mathrm{SE}}^{(\mathrm{i})}-{\mathrm{x}}_{\mathrm{SE}}^{(\mathrm{i}+1)}}{{\mathrm{y}}_{\mathrm{SE}}^{(\mathrm{i}+1)}-{\mathrm{y}}_{\mathrm{SE}}^{(\mathrm{i})}}\cdot  {\mathrm{x}}_{\mathrm{CE}}^{(\mathrm{i})}+\frac{1}{2}\cdot  \left({\mathrm{y}}_{\mathrm{SE}}^{(\mathrm{i})}+{\mathrm{y}}_{\mathrm{SE}}^{(\mathrm{i}+1)}+\frac{{\left({\mathrm{x}}_{\mathrm{SE}}^{(\mathrm{i}+1)}\right)}^{2}-{\left({\mathrm{x}}_{\mathrm{SE}}^{(\mathrm{i})}\right)}^{2}}{{\mathrm{y}}_{\mathrm{SE}}^{(\mathrm{i}+1)}-{\mathrm{y}}_{\mathrm{SE}}^{(\mathrm{i})}}\right)$$

But the coordinates (x_E_^(i)^,y_E_^(i)^) of the curvature point E^(i)^ are:

51 $${\mathrm{x}}_{\mathrm{E}}^{(\mathrm{i})}=\frac{{\mathrm{r}}_{\mathrm{S}}}{{\mathrm{R}}_{\mathrm{CE}}^{(\mathrm{i})}}\cdot  \left({\mathrm{x}}_{\mathrm{CE}}^{(\mathrm{i})}-{\mathrm{x}}_{\mathrm{SE}}^{(\mathrm{i})}\right)+{\mathrm{x}}_{\mathrm{SE}}^{(\mathrm{i})}$$

52 $${\mathrm{y}}_{\mathrm{E}}^{(\mathrm{i})}=\frac{{\mathrm{r}}_{\mathrm{S}}}{{\mathrm{R}}_{\mathrm{CE}}^{(\mathrm{i})}}\cdot  \left({\mathrm{y}}_{\mathrm{CE}}^{(\mathrm{i})}-{\mathrm{y}}_{\mathrm{SE}}^{(\mathrm{i})}\right)+{\mathrm{y}}_{\mathrm{SE}}^{(\mathrm{i})}$$

where:

53 $${\mathrm{R}}_{\mathrm{CE}}^{(\mathrm{i})}=\sqrt{{\left({\mathrm{x}}_{\mathrm{CE}}^{(\mathrm{i})}-{\mathrm{x}}_{\mathrm{SE}}^{(\mathrm{i})}\right)}^{2}+{\left({\mathrm{y}}_{\mathrm{CE}}^{(\mathrm{i})}-{\mathrm{y}}_{\mathrm{SE}}^{(\mathrm{i})}\right)}^{2}}$$

And must be met the following condition:

54 $${\mathrm{y}}_{\mathrm{E}}^{(\mathrm{i}+1)}-{\mathrm{y}}_{\mathrm{E}}^{(\mathrm{i})}>0$$

Then there is no self-interlacing of the curvature pitch line like in Fig. 9.

The mathematical formulas presented above allow calculating the coordinates of the point E on the curvature pitch line only in the range of the angle β_E_. The points in the second half of the curvature hump are a mirror image of the points E with respect to the straight-line k. But the total pitch line of the curvature is the circular array of the hump pitch line with respect to the origin of the coordinate system.

## The length of the rotor pitch line and the curvature pitch line

**Figure 23:**
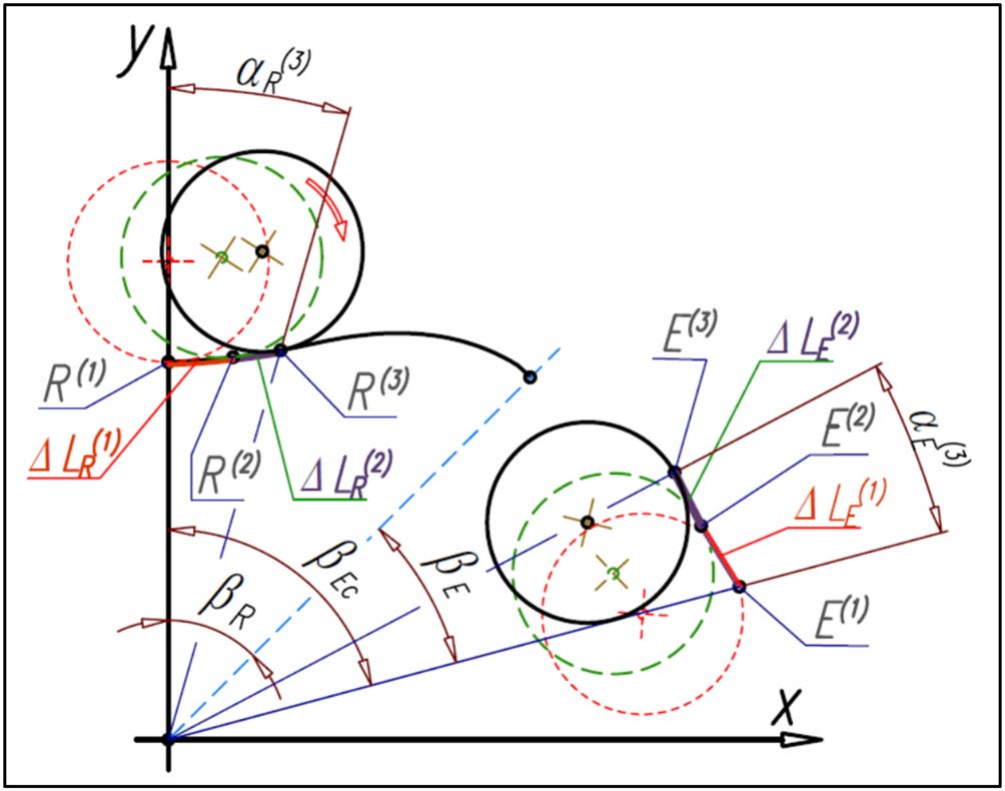
The elementary lengths of the rotor and curvature (∆L_R_^(i)^ and ∆L_E_^(i)^).

The length L_R_ of the rotor pitch line in terms of the angle α_R_ is the sum of the elementary lengths ∆L_R_^(i)^ defined by two adjacent points (R^(i)^ and R^(i+1)^) of the rotor pitch line (Fig. 23), that is:

55 $${\mathrm{L}}_{\mathrm{R}}={\sum }_{i=1}^{n}\sqrt{{\left({\mathrm{x}}_{\mathrm{R}}^{(\mathrm{i}+1)}-{\mathrm{x}}_{\mathrm{R}}^{(\mathrm{i})}\right)}^{2}+{\left({\mathrm{y}}_{\mathrm{R}}^{(\mathrm{i}+1)}-{\mathrm{y}}_{\mathrm{R}}^{(\mathrm{i})}\right)}^{2}}$$

Similarly, the length L_E_ of the curvature pitch line in the range of angle α_E_ is the sum of the elementary lengths ∆L_E_^(i)^ defined by two adjacent points (E^(i)^ and E^(i+1)^) of the curvature pitch line (Figs. 19, 22, 23), that is:

56 $${\mathrm{L}}_{\mathrm{E}}={\sum }_{i=1}^{n}\sqrt{{\left({\mathrm{x}}_{\mathrm{E}}^{(\mathrm{i}+1)}-{\mathrm{x}}_{\mathrm{E}}^{(\mathrm{i})}\right)}^{2}+{\left({\mathrm{y}}_{\mathrm{E}}^{(\mathrm{i}+1)}-{\mathrm{y}}_{\mathrm{E}}^{(\mathrm{i})}\right)}^{2}}$$

If α_R_ = β_R_ then L_R_ = L_Rc_ and if α_E_ = β_E_ then L_E_ = L_Ec_.

## The angle between satellites and the number of satellites

**Figure 24:**
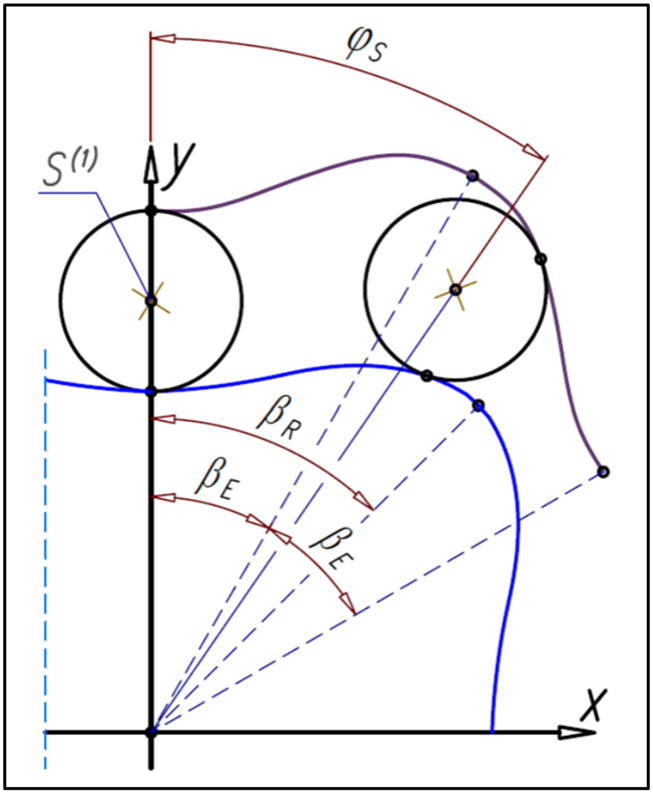
The angle φ_S_ between satellites.

If the angle β_E_ corresponds to curvature hump then the rotate of the curvature by the angle

57 $${\uptheta }_{\mathrm{E}}=2\cdot  {\upbeta }_{\mathrm{E}}$$

will enable the placement of the next satellite in the same place (point S^(1)^—Fig. 24). The formulas (10) and (34) indicate that the angle φ_S_ between satellites is:

58 $${\mathrm{\varphi }}_{\mathrm{S}}=\frac{2\cdot \uppi }{{\mathrm{n}}_{\mathrm{R}}+{\mathrm{n}}_{\mathrm{E}}}$$

The number n_S_ of satellite in mechanism is:

59 $${\mathrm{n}}_{\mathrm{S}}=\frac{2\cdot \uppi }{{\mathrm{\varphi }}_{\mathrm{S}}}={\mathrm{n}}_{\mathrm{R}}+{\mathrm{n}}_{\mathrm{E}}$$

## Types of satellite mechanisms

By the type of satellite mechanism should be understood its characteristic future, i.e. the number n_R_ of the rotor humps and the number n_E_ of the curvature humps. Therefore the type of mechanism is marked as “n_R_ x n_E_”. If the number n_E_ of the curvature humps increases in relation to the number n_R_ of the rotor humps then increases the satellite diameter and decreases the distance between axes of two adjacent satellites. Thus, in a satellite mechanism of any type, each pair of adjacent satellites must satisfy the following condition:

60 $$\sqrt{{\left({\mathrm{x}}_{\mathrm{S}}^{(\mathrm{i}+1)}-{\mathrm{x}}_{\mathrm{S}}^{(\mathrm{i})}\right)}^{2}+{\left({\mathrm{y}}_{\mathrm{S}}^{(\mathrm{i}+1)}-{\mathrm{y}}_{\mathrm{S}}^{(\mathrm{i})}\right)}^{2}}>2\cdot  \left({\mathrm{r}}_{\mathrm{S}}+{\mathrm{h}}_{\mathrm{hS}}\right)$$

where $\left({\mathrm{x}}_{\mathrm{S}}^{(\mathrm{i})},{\mathrm{y}}_{\mathrm{S}}^{(\mathrm{i})}\right)$ and $\left({\mathrm{x}}_{\mathrm{S}}^{(\mathrm{i}+1)},{\mathrm{y}}_{\mathrm{S}}^{(\mathrm{i}+1)}\right)$—coordinates of two adjacent satellites, h_hs_—the tooth head height.

**Figure 25:**
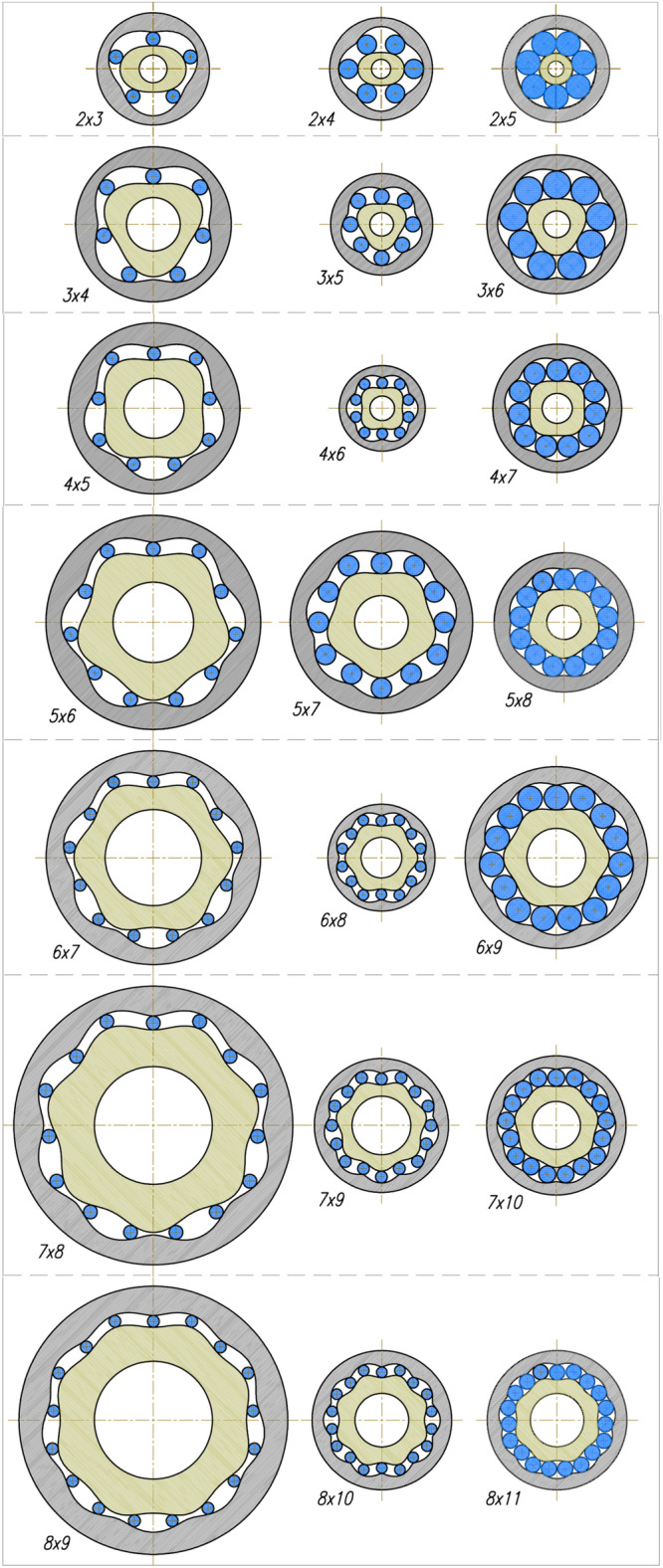
Various types of satellite mechanisms.

The Types of satellite mechanisms that meet the condition (60) are shown in Fig. 25. It should be noted that, regardless of the type of mechanism, the distance between the satellite’s pitch diameters decreases if the difference in the number of rotor and curvature humps increases. Therefore these mechanisms are characterized by a large number of teeth and large dimensions.

Is not possible to build a satellite mechanism if:

61 $${\mathrm{n}}_{\mathrm{E}}-{\mathrm{n}}_{\mathrm{R}}>3$$

## The parameters of satellite mechanism for the cosinusoidal shape of the rotor pitch line

Below were determined the satellite mechanism parameters for the radius r_R_ of the rotor pitch curve expressed as (Fig. 13):

62 $${\mathrm{r}}_{\mathrm{R}}={\mathrm{r}}_{\mathrm{c}}-{\mathrm{L}}_{\mathrm{h}}$$

where:

63 $${\mathrm{L}}_{\mathrm{h}}={\mathrm{A}}_{\mathrm{h}}\cdot  \mathrm{cos}\left({\mathrm{n}}_{\mathrm{R}}\cdot  {\mathrm{\alpha }}_{\mathrm{R}}\right)$$

That is a cosine curve with an amplitude A_h_ is “wound” on the circle with a radius r_c_. The coordinates (x_R_,y_R_) of the point R on the rotor pitch line are:

64 $${\mathrm{x}}_{\mathrm{R}}=\left({\mathrm{r}}_{\mathrm{c}}-{\mathrm{A}}_{\mathrm{h}}\cdot  \mathrm{cos}\left({\mathrm{n}}_{\mathrm{R}}\cdot  {\mathrm{\alpha }}_{\mathrm{R}}\right)\right)\cdot  \mathrm{sin}{\mathrm{\alpha }}_{\mathrm{R}}$$

65 $${\mathrm{y}}_{\mathrm{R}}=\left({\mathrm{r}}_{\mathrm{c}}-{\mathrm{A}}_{\mathrm{h}}\cdot  \mathrm{cos}\left({\mathrm{n}}_{\mathrm{R}}\cdot  {\mathrm{\alpha }}_{\mathrm{R}}\right)\right)\cdot  \mathrm{cos}{\mathrm{\alpha }}_{\mathrm{R}}$$

The slope a_(R)p_ f the straight-line y_(R)p_ perpendicular to the tangent line y_(R)t_ in the point R is:

66 $${\mathrm{a}}_{\left(\mathrm{R}\right)\mathrm{p}}=\frac{{\mathrm{r}}_{\mathrm{c}}\cdot  \mathrm{sin}{\mathrm{\alpha }}_{\mathrm{R}}-{\mathrm{A}}_{\mathrm{h}}\cdot  \left(\mathrm{cos}\left({\mathrm{n}}_{\mathrm{R}}\cdot  {\mathrm{\alpha }}_{\mathrm{R}}\right)\cdot  \mathrm{cos}{\mathrm{\alpha }}_{\mathrm{R}}+\mathrm{sin}\left({\mathrm{n}}_{\mathrm{R}}\cdot  {\mathrm{\alpha }}_{\mathrm{R}}\right)\cdot  \mathrm{sin}{\mathrm{\alpha }}_{\mathrm{R}}\right)}{{\mathrm{r}}_{\mathrm{c}}\cdot  \mathrm{sin}{\mathrm{\alpha }}_{\mathrm{R}}-{\mathrm{A}}_{\mathrm{h}}\cdot  \left(\mathrm{cos}\left({\mathrm{n}}_{\mathrm{R}}\cdot  {\mathrm{\alpha }}_{\mathrm{R}}\right)\cdot  \mathrm{sin}{\mathrm{\alpha }}_{\mathrm{R}}+\mathrm{sin}\left({\mathrm{n}}_{\mathrm{R}}\cdot  {\mathrm{\alpha }}_{\mathrm{R}}\right)\cdot  \mathrm{cos}{\mathrm{\alpha }}_{\mathrm{R}}\right)}$$

Table 1 summarizes the parameters of selected satellite mechanisms calculated using the first method (see “The first method: searching for the perfect solution”). Whereas Table 2 summarizes parameters of selected satellite mechanisms calculated using the second method (see “The second method”), assuming:

- different number of teeth z_Rc_ corresponding to the length L_Rc_ of the rotor pitch line,
- ${\mathrm{y}}_{\mathrm{E}}^{(\mathrm{i}+1)}-{\mathrm{y}}_{\mathrm{E}}^{\left(\mathrm{i}\right)}\approx 0$ that is for A_h_/r_c_ = max.

**Table 1:** Parameters of satellite mechanisms from Fig. 25 calculated according to the first method for tooth module m = 1 mm.

n_R_ [–]	n_E_ [–]	r_c_ [mm]	A_h_ [mm]	z_Rc_ [–]	z_S_ [–]	δ [mm]
2	3	40.37399	5.046749	20.5	20	0.117
	4	18.493198	3.0822	9.5	18	0.0155
	5	403.74774	10.09369	202	606	0.0002
3	4	43.410709	5.601382	15	14	0.0164
	5	21.705355	2.800691	7.5	14	0.0118
	6	66.124829	6.399177	22.5	65	0.0884
4	5	50.076	4.972	13	12	0.048
	6	21.055345	2.263776	5.5	10	0.0465
	7	54.782795	4.113894	14	40	0.103
5	6	67.337161	5.430416	14	13	0.0174
	7	48.673506	3.2449	10	19	0.0166
	8	96.233577	4.436368	19.5	57	0.0006
6	7	69.585737	4.373961	12	11	0.1049
	8	31.594103	2.256722	5.5	10	0.0418
		28.588603	2.154503	5	9	0.0413
	9	93.804061	4.824209	16	46	0.0371
7	8	94.437105	5.312087	14	13	0.004
	9	40.234706	2.445879	6	11	0.0086
	10	75.662193	2.892966	11	32	0.0405
8	9	90.007125	3.375267	11.5	11	0.0035
	10	41.949352	2.359651	5.5	10	0.0037
	11	78.096644	3.075055	7	20	0.0061

**Table 2:** Parameters of selected satellite mechanisms from Fig. 25 calculated according to the second method for A_h_/r_c_ = max (for tooth module m = 1 mm).

n_R_ [–]	n_E_ [–]	r_c_ [mm]	A_h_ [mm]	z_Rc_ [–]	z_S_ [–]	x_smin_ [–]
4	6	17.23564	1.842059	4.5	8	0.132
		19.15098	2.046419	5	9	0.091
		21.066078	2.251061	5.5	10	0.05
		22.981176	2.455703	6	11	0.009
6	8	28.593603	2.150736	5	9	0.049
		31.452963	2.36581	5.5	10	0.004
		34.312323	2.580883	6	11	− 0.041
8	10	38.004438	2.21839	5	9	0.02
		41.804882	2.440229	5.5	10	− 0.027
		45.605326	2.662068	6	11	− 0.075

In both cases the parameters were determined for tooth module m = 1 mm.

Development of satellite mechanisms with n_R_ > 8 is possible but these mechanisms are unlikely to find technical application.

## Verification of satellite mechanism designing method

The parameters of satellite mechanism type 4 × 5, shown in Table 1, after scaling to the module m = 0,5 mm correspond to the parameters of the mechanism shown in Fig. 4. Thus, it confirms the correctness of the design methodology and the performed calculations.

**Figure 26:**
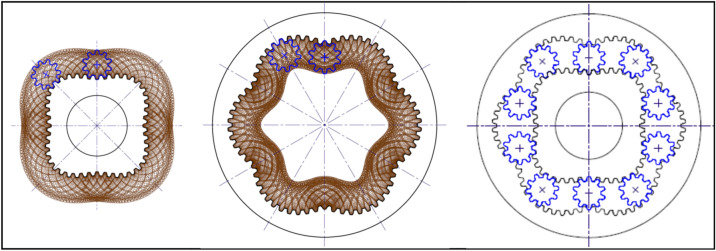
Rotor, curvature and complete satellite mechanism type 4 × 6 (pressure angle 20°, z_S_ = 10, z_Rc_ = 5.5, z_R_ = 44, z_E_ = 66).

**Figure 27:**
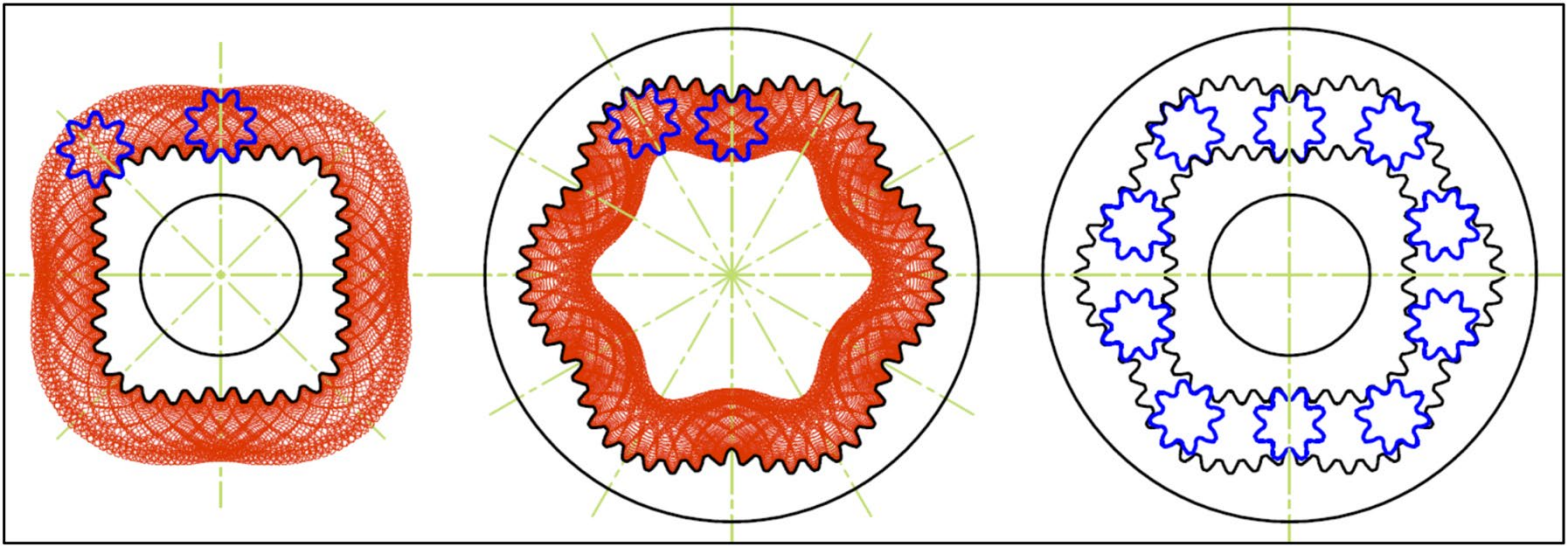
Rotor, curvature and complete satellite mechanism type 4 × 6 (pressure angle 30°, z_S_ = 8, z_Rc_ = 4.5, z_R_ = 36, z_E_ = 54).

**Figure 28:**
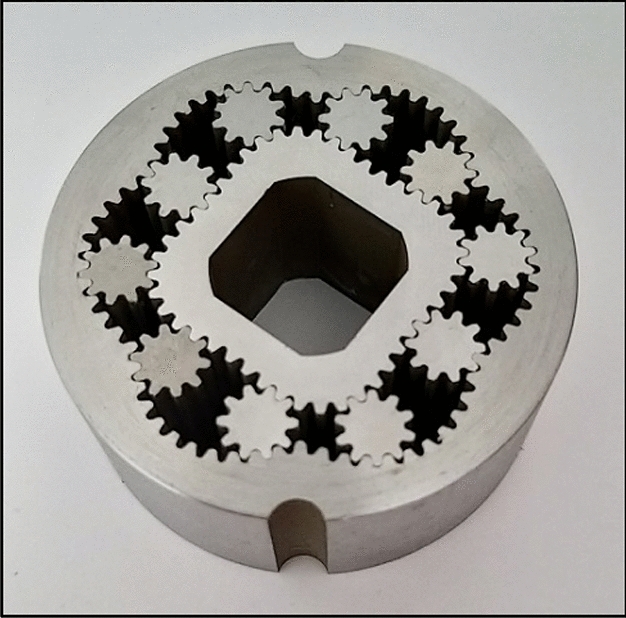
Satellite mechanism type 4 × 6: made of metal by WEDM (z_S_ = 10, z_Rc_ = 5.5, z_R_ = 44, z_E_ = 66).

In order to verify the presented procedure of designing, a sample satellite mechanism was designed, manufactured and examined. The projects of rotor and curvature, created according to the above-described methodology are presented in Figs. 26 and 27. But Fig. 28 shows satellite mechanism type 4 × 6 made of metal by WEDM.

The satellite mechanism was checked for smooth rotation.

## Summary

This article presents the method of designing a satellite mechanism based on the adopted function of the rotor pitch line. The sequence of the proceedings for selecting the parameters of the satellite mechanism is described. All technically possible types of satellite mechanisms are also presented. These mechanisms were calculated assuming the sinusoidal function to determine the shape of the rotor pitch line. From the point of view of the construction of hydraulic displacement machines, the use of a rotor with a circular-sinusoidal shape is justified.

Both the shape of the rotor teeth and the curvature teeth are determined by the shape of the satellite teeth. The satellite is a gear-shaper cutter. In this way the teeth interference was eliminated. This is an undoubted advantage of the presented method. Furthermore, the presented method of design allows to avoid the self-intersection of curvature pitch line and undercutting the curvature teeth. Is possible to apply a teeth correction and to create a satellite mechanism even with a very small number of satellite teeth (for example z_S_ = 8).

The practical verification shown that using the presented method of design has good results. In the manufactured mechanism, no problems with gears meshing were observed—the mechanism worked smoothly, without jamming.

The results presented in this paper can constitute fundamentals for further investigations of other particular properties of different types of satellite mechanisms, such as:

- the volume of the working chamber as a function of the angle of rotation of the rotor or curvature. The working chamber should be understood as a volume formed by two adjacent satellites, rotor and curvature;
- geometrical displacement of a hydraulic machine with different types of satellite mechanisms;
- theoretical characteristics of torque and flow rate in a mechanism and thus an irregularity of flow rate and torque also;
- satellite kinematics and dynamics;
- mechanical losses.

Undoubtedly, an important issue will be the analysis of the impact of tooth structure (e.g. involute teeth, circular-arc teeth, etc.) on their strength.

Nevertheless, the methodology of designing the curvature is universal for different shapes of the rotor. The methodology of designing the satellite mechanism, presented below, enables their manufacturing using the wire electrical discharge machining method.

## Data Availability

The datasets generated and/or analysed during the current study are not publicly available due to patent protection of the solutions presented in the article (patent applications P.437749, P.437750 and P.437751) but are available from the corresponding author on reasonable request.
